# Achieving Photo‐Activated Circularly Polarized Room Temperature Phosphorescence from Natural Biopolymers

**DOI:** 10.1002/advs.202523073

**Published:** 2026-01-11

**Authors:** Shaoyi Cao, Mingcong Xu, Bang An, Baoqi Li, Wenye Sun, Wenbo Cui, Rui Teng, Chunhui Ma, Sha Luo, Bing Tian, Zhijun Chen, Shouxin Liu, Wei Li

**Affiliations:** ^1^ State Key Laboratory of Woody Oil Resources Utilization Northeast Forestry University Harbin China; ^2^ Key Laboratory of Bio‐based Material Science and Technology of Ministry of Education Northeast Forestry University Harbin China

**Keywords:** chirality tunable, circularly polarized room temperature phosphorescence, hydroxypropyl cellulose, multi‐color, photo‐activation

## Abstract

Developing sustainable photo‐activated circularly polarized room temperature phosphorescent (CPRTP) materials is attractive for optoelectronic applications while are difficult to achieve. Here, we report the first example of bio‐based photo‐activated CPRTP material by anchoring arylboronic acids into hydroxypropyl cellulose (HPC) matrix via B─O covalent bonding. The rigid environment provided by B─O covalent bonds and hydrogen bonds stabilizes the triplet excitons, the residual oxygen is consumed upon continuous UV light irradiation, enabling photo‐activated CPRTP with multi‐color, high‐dissymmetry factor (g_l__um_ up to ‐0.43) and prolonged lifetime from 0.22 ms to 1.57 s. More interestingly, by controlling the drying kinetics, HPC films exhibit tunable and dynamic switchable CPRTP with opposite handedness. In addition, due to the water sensitive phosphorescent nature, HPC films also show a responsive on/off CPRTP under cycled water/heat stimuli treatment. Based on the moldable and responsive CPRTP properties of the HPC based materials, the application of information photo‐controlled encrypted tags, wavelength‐dependent phosphorescent decorated patterns, multi‐mode afterglow inks have been successfully demonstrated. This study offers new insights into the intrinsic chiral luminescence of cellulose macromolecules, providing a sustainable platform for the efficient design and functional application of photo‐activated CPRTP materials.

## Graphical abstract

This bio‐based chiral luminescent system exhibit photo‐activated CPRTP by anchoring arylboronic acids to hydroxypropyl cellulose (HPC) matrix. Notably, by controlling the drying kinetics, dynamic switchable CPRTP with opposite handedness can be obtained due to a balance of photonic bandgap (PBG) effect and chirality transfer effect induced by the HPC photonic structures. Leveraging the abundant and responsive CPRTP features, light‐controlled information storage, encryption tags, and multimode afterglow inks are well showcased.

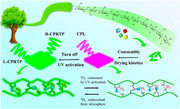

## 1 Introduction

Room temperature phosphorescent (RTP) materials, as a new generation of optical materials, are widely used in the fields of information storage [1, 2, 3, 4, 5], scintillators [6], chemo‐sensors [7], flexible displays [8, 9], and medical diagnostics [10, 11, 12]. Especially, in order to broaden the optical information dimension of RTP materials, efforts have been devoted by imparting chirality to RTP system to build circularly polarized room temperature phosphorescent (CPRTP) materials [13, 14, 15, 16, 17, 18, 19, 20, 21, 22, 23], which is beneficial for the advanced applications such as grayscale afterglow imaging [24], circularly polarized OLEDs [25, 26, 27, 28], and multi‐level anti‐counterfeiting [29, 30, 31, 32, 33, 34, 35]. Recently, in addition to the optimization of color tuning, lifetime, brightness, and quantum yield, the stimuli‐responsive CPRTP platform emerged rapidly and attracting considerable research interest due to their abundant and dynamic tunable chiroptical information [36, 37, 38, 39].

Light stimulation offers remarkable advantages for stimuli‐responsive CPRTP materials, owing to its remote, precise, and contact‐free controllability. To date, two primary strategies have been proposed to achieve photo‐responsive CPRTP, though only a limited number of successful examples have been reported. The first approach involves the design of systems integrating both RTP centers and photo‐responsive helical structures [40, 41]. However, this strategy remains challenging due to the complexity of the required chemical synthesis. In contrast, the second strategy combines photo‐activated RTP luminophores with chiral helical templates, offering a more feasible route to achieve photo‐responsive CPRTP [42]. Photo‐activated CPRTP, an emerging class of photo‐responsive systems, has witnessed notable progress. Researchers have successfully achieved photo‐activated CPRTP emission with a high dissymmetry factor (g_l__um_) by doping chiral phosphorescent chromophores into rigid polymer systems or by bilayer architectures assembly, advancing their potential optoelectronic applications [15, 18, 43, 44]. However, several critical challenges impede their practical implementation. The synthesis of these materials typically involves complex, multi‐step procedures that hinder reproducibility and scalability. Furthermore, the predominant reliance on petroleum‐derived polymers raises concerns regarding environmental sustainability and long‐term viability. Consequently, developing a facile and sustainable strategy to construct photoactivated CPRTP materials represents a significant challenge and an urgent priority.

Cellulose, as the most abundant natural bio‐polymer, the intrinsic chiral structure and functionalize hydroxyl sites provide an ideal platform for the sustainable construction of CPRTP materials [45, 46, 47, 48, 49, 50, 51]. Through a simple host‐guest doping strategy [52, 53], researchers have introduced achiral luminophores into cellulose derivatives to prepare bio‐based CPRTP materials with long lifetime and tunable colors, via the hydrogen‐bonding interactions and intrinsic chirality transfer from cellulose derivatives. Additionally, leveraging the intrinsic photonic bandgap (PBG) effect of cellulose nanocrystals (CNCs), a co‐assembly strategy has been successfully employed to fabricate CPRTP materials, demonstrating right‐handed circularly polarized emission, long lifetime, and high g_l__um_ values [54, 55, 56, 57, 58]. In addition to the excellent chiroptical properties, cellulose based CPRTP materials can be completely degraded in soil without burdening the environment. However, to the best of our knowledge, the intrinsic photoactivated CPRTP still remains unexploited from these bio‐based cellulose materials.

**FIGURE 1:**
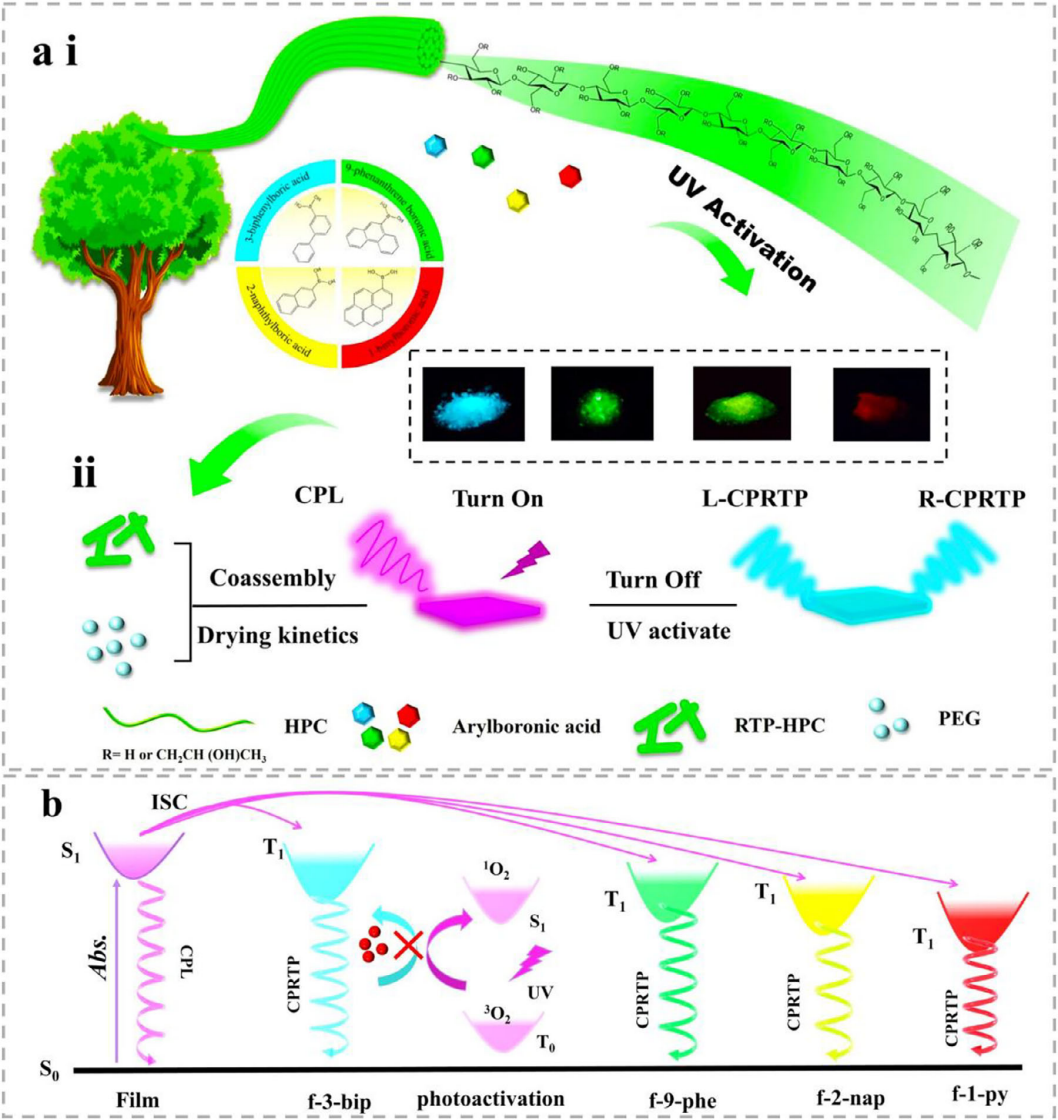
Fabrication of cellulose‐based photo‐activated CPRTP materials. (ai) Schematic illustration of the RTP‐HPC powder preparation process by a click chemistry reaction between HPC and arylboronic acids. (aii) Design strategy for constructing HPC films with both CPL and chirality tunable photo‐activated CPRTP by controlling the drying kinetics. (b) Schematic diagram of multi‐color photo‐activated CPRTP mechanism in HPC film under UV irradiation.

In this work, we report the first bio‐based photoactivated CPRTP material, using a B─O click reaction strategy on hydroxypropyl cellulose (HPC) macromolecules (Figure 1). Arylboronic acids with different conjugate degrees were riveted to HPC by B─O covalent bonding reaction, thus promoting photoactivated RTP with a wide range of afterglow colors (Figure 1ai). At the same time, chiral photonic HPC films were made from RTP‐HPC, realizing the simultaneous emission of circularly polarized luminescence (CPL) and CPRTP. In addition, by controlling the drying kinetics of RTP‐HPC, left‐handed (L‐) CPRTP and right‐handed (R‐) CPRTP emission was achieved simultaneously (Figure 1aii). Under continuous UV irradiation, this residual oxygen of RTP‐HPC is progressively consumed by triplet excitons, thereby activating persistent CPRTP emission with multi‐color afterglow and enhanced lifetime (Figure 1b). Furthermore, we investigated the stimuli‐responsive CPRTP of HPC films over multiple cycles of humidity/heat treatment. Based on the moldable and photo‐programmable CPRTP properties, RTP‐HPC can be applied for use in information security, phosphorescent decorations, and afterglow ink applications, which is environmentally friendly and has great commercialization prospects.

## 2 Results and Discussion

### 2.1 Photoactivated Properties of RTP‐HPC

**FIGURE 2:**
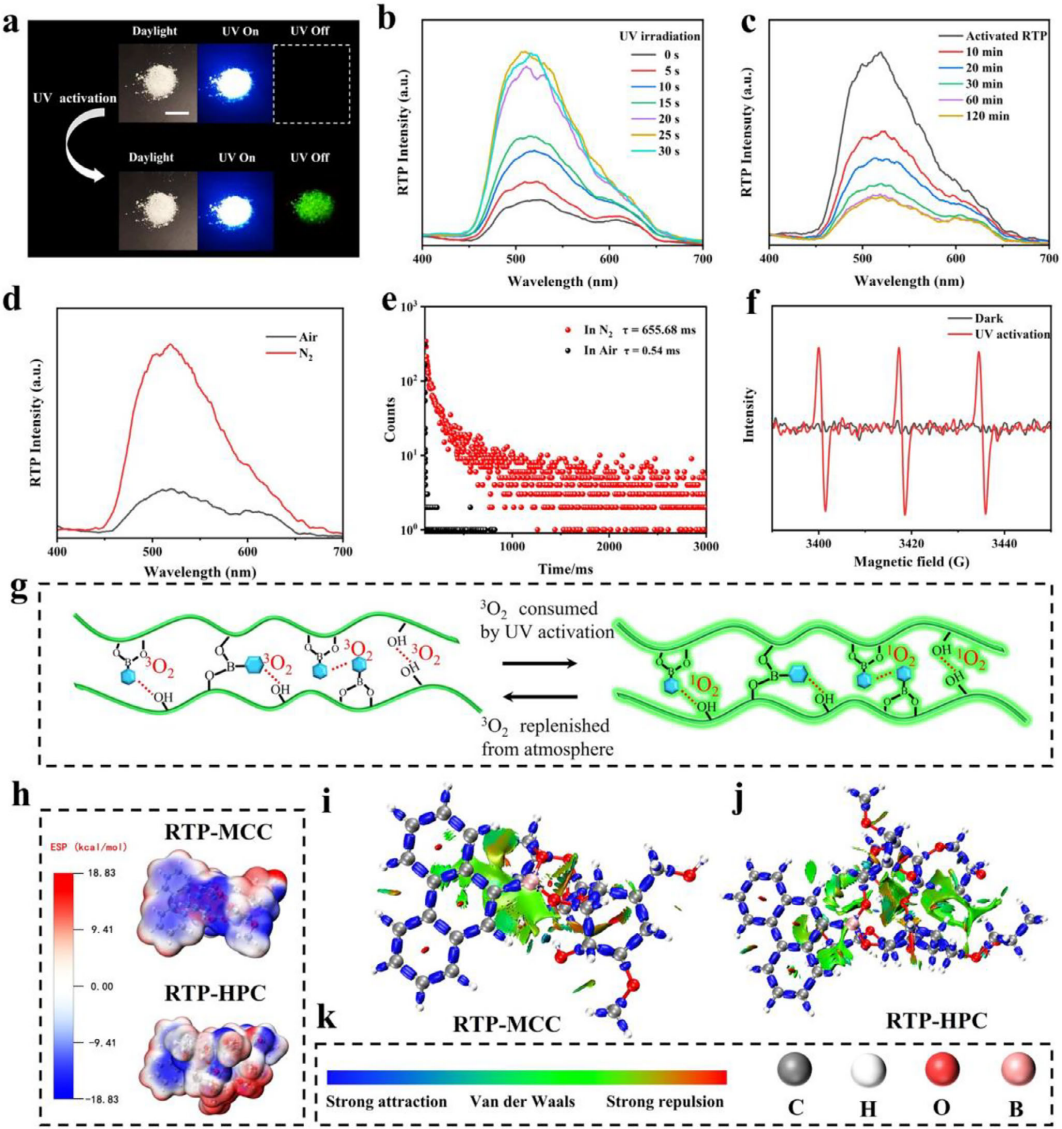
The photoreactivation property of RTP‐HPC. (a) Images of RTP‐HPC upon UV (310 nm) irradiation and after removing the UV excitation (upper), Images of UV‐activated RTP‐HPC upon UV irradiation and after removing the UV excitation (lower), scale bar = 1 cm. (b) Phosphorescence spectra of RTP‐HPC at different irradiation times. (c) Phosphorescence spectra of RTP‐HPC upon UV irradiation and phosphorescence spectra at different times in the environment. (d) Phosphorescence spectra and (e) Comparison of phosphorescence lifetimes of RTP‐HPC in air and N_2_ without UV activation. (f) EPR spectra of RTP‐HPC in the presence of TEMP in the dark and upon UV irradiation. (g) The scheme of photo‐activated RTP‐HPC. (h) ESP distribution of RTP‐MCC and RTP‐HPC. (i–k) IGMH analysis of the intermolecular interactions of RTP‐MCC and RTP‐HPC.

In the presence of deionized water and ammonium hydroxide, a click chemistry reaction between HPC and arylboronic acids was used to promote the formation of RTP‐HPC p‐3‐bip, p‐2‐nap, p‐9‐phe, and p‐1‐py. Taking p‐9‐phe as an example, we first investigated the effect of arylboronic acid doping concentration (0.1, 0.2, 0.5, 1.0, 2.0 wt.%) on the phosphorescence of RTP‐HPC. The p‐9‐phe's phosphorescence intensity showed a tendency to increase and then decrease with the increase of the doping concentration, and reached the maximum at the doping concentration of 0.5 wt.%, so the optimal doping concentration of arylboronic acid was about 0.5 wt.% (Figure S1). This suggests that too much organic phosphor cannot be efficiently located and confined by the HPC matrix, resulting in energy dissipation and triplet quenching [59]. Notably, p‐9‐phe exhibited significant photoactivation of RTP properties. Initially, p‐9‐phe exhibited a weak RTP that is almost impossible to observe. After 5 s of UV (310 nm) irradiation, the RTP intensity increased, and its RTP emission showed a continuous enhancement as the irradiation time further increased. After 30 s exposure to UV light, the RTP intensity increased by 4 times compared with the original, and the RTP lifetime also increased from 0.54 to 1481 ms (Figure 2a,b; Figure S2 and Video S1). Interestingly, the photo‐activated RTP emission of p‐9‐phe in the air environment decreased after the UV light source was removed. After 2 h, RTP emissions almost returned to the initial weak state (Figure 2c). Furthermore, after 10 cycles, the RTP intensity of p‐9‐phe demonstrated no attenuation, demonstrating excellent stability (Figure S3). Similarly, the RTP of p‐2‐nap, p‐9‐phe, and p‐1‐py also showed obvious photoactivation properties, and their lifetime was significantly increased after UV activation (Figures S4–S6). This particular photoactivation phenomenon has never been reported in cellulose‐based RTP materials.

To elucidate the photoactivation mechanism of the RTP‐HPC, we compared the phosphorescent behavior of the model compound p‐9‐phe under air and N_2_. Under a N_2_ environment, p‐9‐phe exhibited significantly enhanced initial RTP performance, featuring a prolonged lifetime of 655.68 ms, which starkly contrasts with its state in air (Figure 2d‐e). To directly probe the role of oxygen for photo‐activated RTP, we conducted electron paramagnetic resonance (EPR) spectroscopy. While samples in the dark showed negligible EPR signals, a strong characteristic signal emerged after UV irradiation in air (Figure 2f). As a control experiment, no significant changes were detected during irradiation in N_2_ (Figure S7). The EPR results further elucidate the mechanism behind the RTP‐HPC photoactivation phenomenon: residual O_2_ in RTP‐HPC is consumed and converted into singlet state oxygen (^1^O_2_) after UV activation, thereby producing the photoactivated RTP performance (Figure 2g) [60].

To further clarify the photo‐activated RTP mechanism, the molecular structure of HPC was investigated. We speculate that the introduction of hydroxypropyl groups onto the natural cellulose backbone partially disrupts the native, dense hydrogen‐bonding networks. This structural modification consequently compromises the material's oxygen barrier properties, allowing residual O_2_ to permeate the matrix and efficiently quench the triplet excitons, resulting in weak initial RTP. To validate our hypothesis, a control sample was fabricated under identical conditions by substituting HPC with microcrystalline cellulose (MCC), which maintains an extensive hydrogen‐bond network. As anticipated, the RTP‐MCC composite exhibited intense RTP immediately after preparation without UV pre‐activation (Figure S8a,b; Video S2), directly demonstrating the superior oxygen barrier effect conferred by MCC's robust hydrogen bonding (Figure S8c). This conclusion was further corroborated by complementary experimental evidence. Fourier transform infrared (FTIR) spectroscopy revealed a broader hydroxyl stretching band (3300–3500 cm^−^^1^) for MCC, which was also shifted to lower wavenumbers compared to HPC, confirming stronger intermolecular hydrogen bonding (Figure S9). XRD analysis (Figure S10) revealed that the crystalline structure of natural cellulose of HPC was partially disrupted, this structural changing consequently resulted in a weakened confinement effect within the HPC matrix. Ground‐state density functional theory (DFT) calculations were used to verify the structure induced RTP difference on RTP‐MCC and RTP‐HPC. Electrostatic surface potential (ESP) analysis identified distinct complementary electrostatic regions in both RTP systems, indicating strong intermolecular hydrogen bonding that establishes a rigid matrix favorable for phosphorescence emission (Figure 2h). Further visualization of noncovalent interactions was achieved through the independent gradient model based on Hirshfeld partition (IGMH). As illustrated in Figure 2i–k, hydrogen bonds (blue isosurfaces) and van der Waals interactions (green isosurfaces) are present in both materials. Remarkably, RTP‐MCC exhibits more intense and spatially concentrated van der Waals forces relative to RTP‐HPC, implying denser molecular packing that effectively restricts oxygen diffusion into the matrix. The relatively weak interaction between HPC and chromophore is responsible for the generation of photoactivated RTP.

### 2.2 Photophysical Properties of RTP‐HPC

**FIGURE 3:**
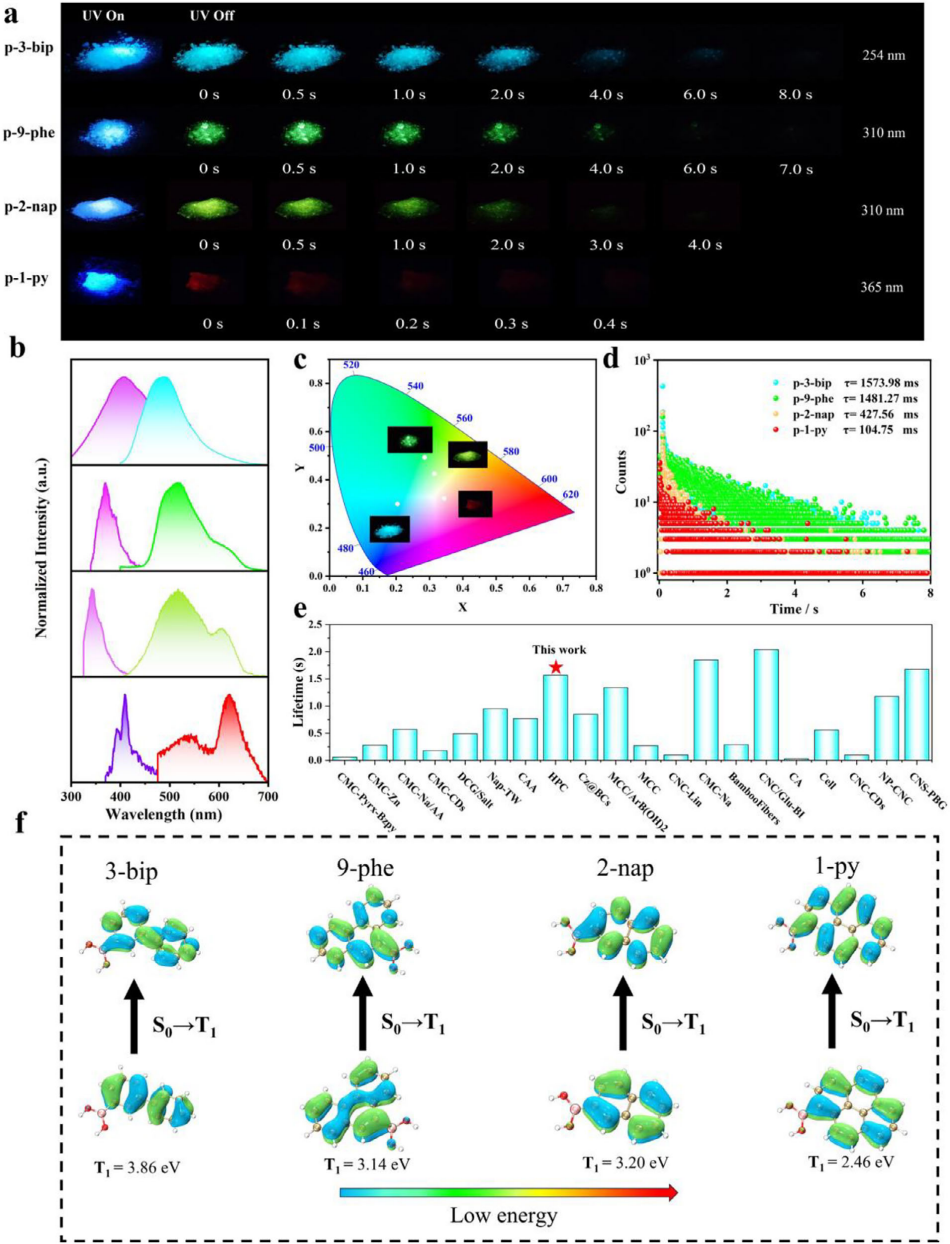
Photophysical properties of RTP‐HPC. (a) Photographs of p‐3‐bip (𝜆ex = 254 nm), p‐2‐nap (𝜆ex = 310 nm), p‐9‐phe (𝜆ex = 310 nm), and p‐1‐py (𝜆ex = 365 nm) before and after UV irradiation for 30 s. (b) Fluorescence and phosphorescence spectra of p‐3‐bip, p‐2‐nap, p‐9‐phe, and p‐1‐py. (c) CIE coordinate plots of p‐3‐bip, p‐2‐nap, p‐9‐phe, and p‐1‐py. (d) Phosphorescence lifetime plots of p‐3‐bip, p‐2‐nap, p‐9‐phe, and p‐1‐py. Insect is an afterglow photo of p‐3‐bip, p‐2‐nap, p‐9‐phe, and p‐1‐py. d) Comparison of phosphorescence lifetimes (after 30 s of UV irradiation) of p‐3‐bip, p‐2‐nap, p‐9‐phe, and p‐1‐py. (e) Comparison of RTP lifetimes between p‐3‐bip and the other reported materials based on cellulose. (f) The theoretical calculations about natural transition orbitals of T_1_ states for 3‐bip, 2‐nap, 9‐phe, and 1‐py.

Under UV irradiation, all the RTP‐HPC samples exhibited white and blue fluorescence with emission peaks located between 350–420 nm. After 30 s of UV activation, RTP‐HPC exhibited clear afterglow with the colors from blue, green to red. Notably, the afterglow time of p‐3‐bip could reach to 8 s observed by the naked eye. As the conjugation degree of the arylboronic acids gradually increased, phosphorescence emission peaks redshifted from 490 to 515 nm, 517, and 623 nm (Figure 3a,b). The International Commission on Illumination (CIE) coordinate charts of RTP‐HPC calculated from the phosphorescence profiles were well consistent with their distinct afterglow colors. (Figure 3c). As shown in the time‐resolved RTP decay curves of RTP‐HPC, p‐3‐bip showed a clearly enhanced phosphorescence lifetime changing from 0.22 to 1573 ms after UV activation (Figure 3d), which is superior to most cellulose‐based RTP materials (Figure 3e). The corresponding phosphorescence lifetimes of p‐2‐nap, p‐9‐phe, and p‐1‐py were 427.56, 1481.27, and 104.75 ms, respectively, which were all significantly increased compared with those samples before photoactivation (Figure 3d).

Furthermore, we investigated the multi‐color photoactivated RTP mechanism of these RTP‐HPC materials. We discovered that the four arylboric acids themselves do not emit RTP because the phosphorescent chromophores exhibit an aggregation‐caused quenching effect in solids (Figure S11) [61, 62]. Once the arylboric acid dispersed in the HPC matrix, the radiative transition channel was activated. The phosphoretic spectrum of RTP‐HPC at room temperature shows emission bands similar to that of arylboric acid in tetrahydrofuran (THF) solution (10^−5^ m) at 77 K (Figure S12). Therefore, the RTP can be attributed to the inherent molecular phosphorescence of single arylboronic acids. It is noteworthy that the phosphorescence spectra of RTP‐HPC are slightly different from those of arylboronic acids molecules in THF solution at 77 K. This discrepancy may be attributed to the distorted conformational change of the arylboronic acid chromophore, which is caused by the strong hydrogen bonding interaction between arylboronic acid and HPC [63]. Four additional RTP‐HPC powders were prepared without adding ammonium hydroxide during the covalent reaction process as controls, they were namely as p‐3‐bip‐m, p‐2‐nap‐m, p‐9‐phe‐m, and p‐1‐py‐m, respectively. As expected, the phosphorescence intensities of these samples were lower than base‐added RTP‐HPC (Figures S13 and S14). The phosphorescence discrepancy may be attributable to the presence of base facilitates the formation of B─O covalent bonds between arylboronic acid and HPC (Figure S15) [64], resulting in further restriction of phosphorescent chromophores, thereby leading to enhanced phosphorescence of the RTP‐HPC. The UV–vis absorption spectra of these RTP‐HPC powders were used to clarify the phosphorescence changes. When the base was added to the catalyzed reaction, the UV–vis absorption spectra of RTP‐HPC showed redshifts compared to the RTP‐HPC without base (Figure S16). The redshifts in the UV–vis absorption spectra might also be attributed to the presence of B─O covalent bonds [65].

Fourier transform infrared (FT‐IR) spectroscopy and X‐ray photoelectron spectroscopy (XPS) provided evidence for the formation of B─O covalent bonds in RTP‐HPC. RTP‐HPC powder (named p‐1‐py‐h) was prepared by reacting to 1‐binylboronic acid and HPC (mass ratio 20 wt.%). The characteristic peak of p‐1‐py‐h at 1073 cm^−1^ can be attributed to the B─O─C group. In addition, two peaks were observed at 667 and 1508 cm^−1^, corresponding to the deformation vibration of the B─OH and the stretching vibration of B─O (Figure S17) [66, 67, 68]. The C1s and B1s curves from XPS (Figure S18) showed covalent C─O─B bonds at 287.6 eV and B─O bonds at 199.43 eV, respectively, which is consistent with the FTIR results [69]. These results suggest that a large number of B─O covalent bonds are present in the base‐catalyzed RTP‐HPC, which is responsible for the long‐lived phosphorescence. The formed B─O covalent bond between the HPC and boronic acid effectively restricted molecular motion of phosphorescent chromophores and suppressed the non‐radiative decay of excited triplet excitons, leading to enhanced RTP with longer lifetime. In order to gain a deeper understanding of multi‐color photoactivated RTP mechanism, theoretical calculations have been carried out (Figure 3f). As depicted in the natural leptonic orbits of the four arylboronic acids, it could be found that the energy of T_1_ decreases with the expansion of π‐conjugation, which is consistent with their experimental redshifted RTP emission. Furthermore, as the energy gap between T_1_ and S_0_ become smaller in these phosphorescent chromophores, the nonradiative leaps of the triplet excitons increase, leading to a redshifted phosphorescence and shorter RTP lifetime.

### 2.3 Full‐Color Tunable CPRTP from HPC

**FIGURE 4:**
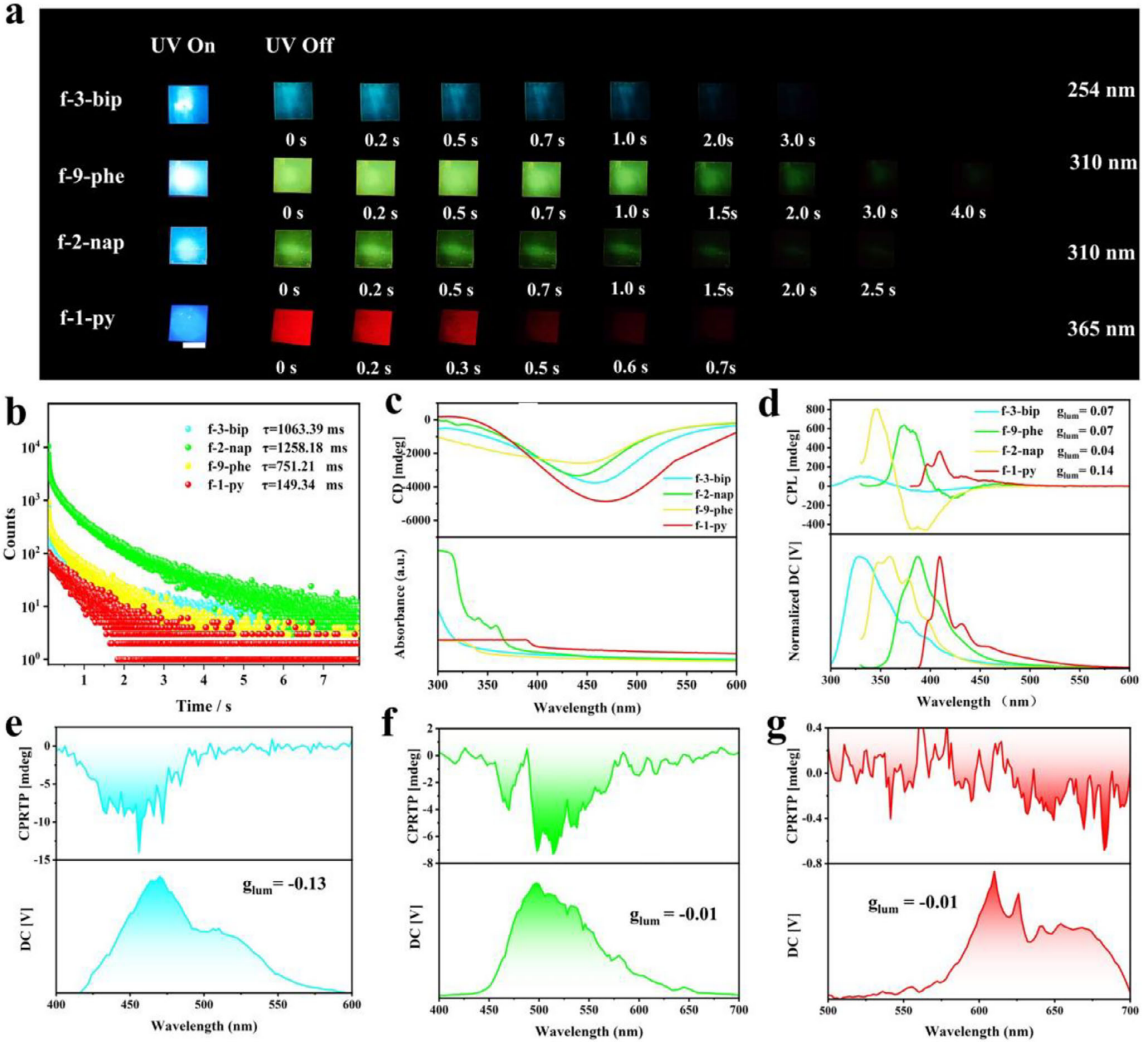
CPL optical properties of RTP‐HPC films. (a) Photographs of f‐3‐bip (𝜆ex = 254 nm), f‐2‐nap (𝜆ex = 310 nm), f‐9‐phe (𝜆ex = 310 nm), and f‐1‐py (𝜆ex = 365 nm) after irradiation for 30 s with different UV excitations, scale bar = 1 cm. (b) Comparison of phosphorescence lifetimes (after 30 s of irradiation from excitation light); (c) CD spectra; (d) CPL spectra of RTP‐HPC films. The CPRTP spectra of (e) f‐3‐bip (f) f‐9‐phe, and (g) f‐1‐py.

Next, we investigate the chiroptical performance of RTP‐HPC films. As illustrated in Figure 4a, these films exhibit distinct phosphorescence emission. Under excitation light, these RTP‐HPC films displayed blue fluorescence, which transitioned to a blue‐to‐red afterglow upon cessation of the excitation source. Notably, the fluorescence and phosphorescence emission of the RTP‐HPC films were similar to their powders (Figure S19). Among them, f‐9‐phe demonstrated the longest afterglow duration of approximately 4 s, followed by f‐3‐bip (3.5 s), f‐2‐nap (2.0 s), and f‐1‐py (0.7 s). This trend in afterglow duration was consistent with the measured phosphorescence lifetimes, where f‐9‐phe exhibited the longest lifetime of 1258.18 ms, with the others decreasing sequentially (Figure 4b).

SEM of all RTP‐HPC films (Figure S20) showed a clear laminar structure, suggesting that HPC immobilized the chirality in the solid film after drying. Circular dichroism (CD) spectra revealed a negative Cotton effect in the RTP‐HPC films, consistent with prior studies demonstrating the right‐handed helical structure of HPC (Figure 4c). The CD spectra of four arylboronic acids did not have obvious peaks, which confirms that the chirality of the RTP‐HPC films originates from the HPC backbone (Figure S21). Subsequently, CPL properties of the RTP‐HPC films was investigated. The CPL spectra of the RTP‐HPC films exhibited positive CPL signals, indicating L‐CPL emission (Figure 4d). To quantify the CPL intensity, the dissymmetry factor (g_lum_) was calculated using the following equation:

$$g_{\mathrm{lum}}\;=2\times \left(I_{L}-I_{R}\right)/\left(I_{L}+I_{R}\right)$$

where I_L_ and I_R_ represent the intensities of L‐CPL and R‐CPL, respectively. The g_lum_ for f‐3‐bip, f‐9‐phe, f‐2‐nap, and f‐1‐py were determined to be 0.07, 0.07, 0.04, and 0.14, respectively, corresponding to the L‐CPL emission signals observed in the CPL spectra (Figure 4d, Figure S22). Interestingly, while the CPL spectra exhibited positive signals, the RTP‐HPC films displayed negative CPRTP signals, with g_lum_ values consistent with those of the CPRTP spectra (Figure 4e‐g; Figures S23 and S24). We speculate that the right‐handed CPRTP arises from the successful chirality transfer from HPC matrix to the non‐chiral phosphorescent guests. In addition, full‐color tunable CPRTP can be achieved in these RTP‐HPC films.

### 2.4 Chirality Tunable CPRTP

Next, to modulate the g_lum_ and handedness of CPRTP, the drying kinetics experiments of RTP‐HPC was systematic conducted. Films prepared from pure HPC solutions require high drying temperatures to achieve structural colors across the visible spectrum. At a dry temperature of 100°C, the HPC films exhibited only a blue color, with a CD peak centered at approximately 420 nm (Figure S25). This limited spectral range hindered the broad band modulation of their chiroptical properties. To address this limitation, we introduced PEG into the HPC matrix, forming physically crosslinked networks. By controlling the drying kinetics, we were able to fix the solid‐state pitch of the films, enabling selective reflection of light across the entire visible spectrum. Following the incorporation of PEG, the observed afterglow colors of g‐3‐bip, g‐9‐phe, g‐2‐nap, and g‐1‐py were blue, yellow, green, and red, respectively, which almost unchanged from the original. The afterglow durations were determined to be 3 s, 4 s, 2.5 s, and 0.7 s, respectively (Figure S26).

**FIGURE 5:**
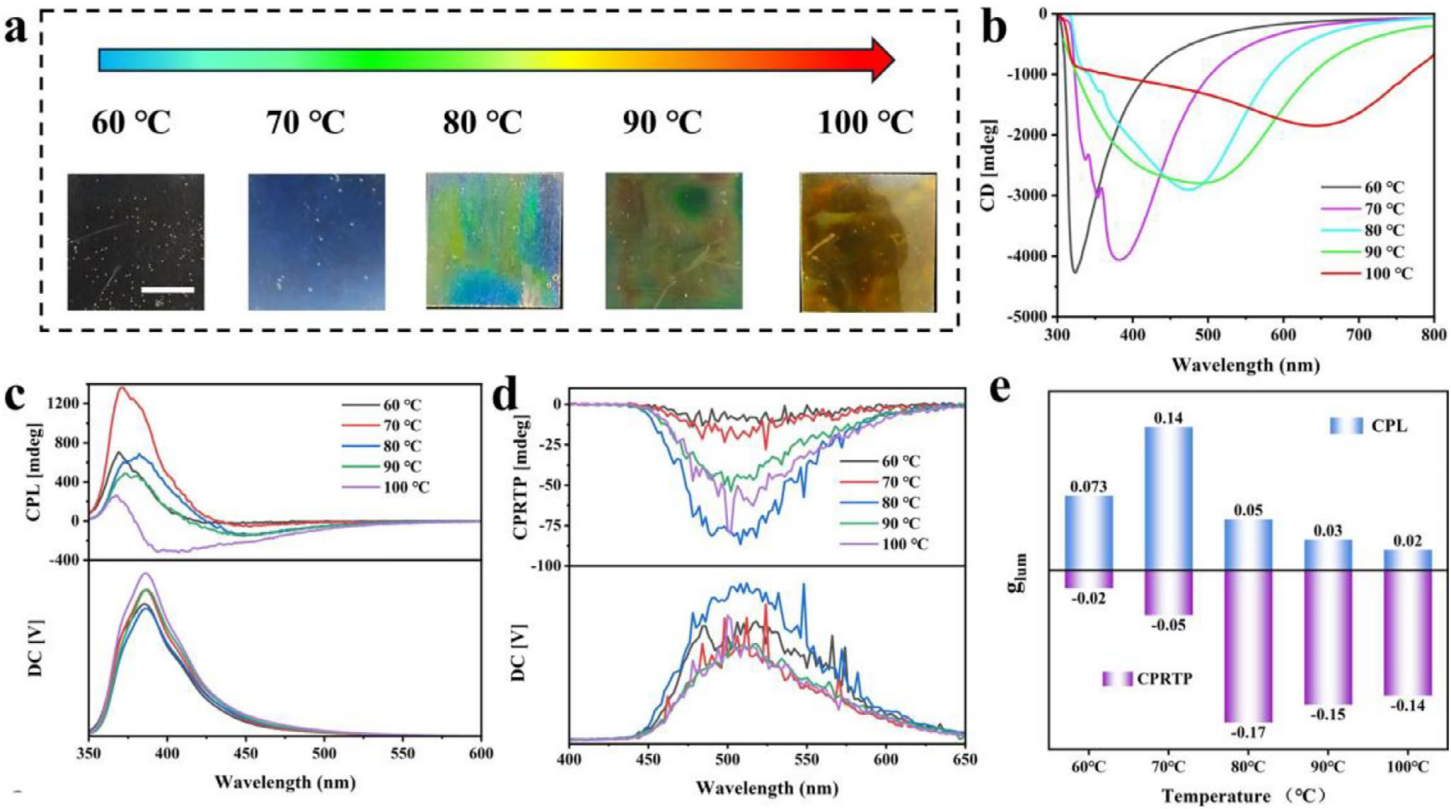
Modulation of chiroptical properties of RTP‐HPC films. (a) corresponding photographs, scale bar = 1 cm; (b) CD spectra of g‐9‐phe dried at 60°C, 70°C, 80°C, 90°C, and 100°C, respectively. (c) CPL spectra; (d) CPRTP spectra; (e) g_lum_ of g‐9‐phe at 60°C; 70°C; 80°C; 90°C; and 100°C (after 30 s of irradiation from UV light respectively).

Using g‐9‐phe as a representative example, we systematically investigated the temperature‐dependent modulation of structural color and chiroptical properties. As illustrated in Figure 5a,g‐9‐phe films transitioned from colorless to red when varying at the drying temperatures from 60°C to 100°C. CD spectra (Figure 5b) exhibited a negative Cotton effect, confirming that the addition of PEG did not alter the right‐handed chiral nematic microstructure of these films. Meanwhile, SEM illustrate that the addition of PEG did not disrupt the lamellar structure of the films, indicating that the films remain their chiral structures (Figure S27). However, the intensity of the CD peaks gradually decreased with increasing temperature. This behavior can be attributed to that at lower temperatures, slow drying rates facilitate the formation of a long‐range ordered chiral nematic structure, whereas higher temperatures induce rapid drying, resulting in a short‐range disordered preference in chiral nematic arrangement [70]. This structural evolution significantly influenced the CPL properties of the films. The CPL spectra revealed both positive and negative signals in g‐9‐phe at different temperatures, indicating the coexistence of R‐CPL and L‐CPL (Figure 5c). This phenomenon arises from the competition between PBG effects and chiral transfer mechanisms. At lower temperatures, the long‐range ordered chiral helical structure enhances the PBG effect, which dominates the L‐CPL emission. Conversely, at higher temperatures, the short‐range disordered chiral nematic structure enhances the chirality transfer effect, thereby resulting in R‐CPL emission. The L‐CPL intensity exhibited a non‐monotonic dependence on temperature, initially increasing and then decreasing, with a maximum observed at 70°C. The g_lum_ exhibited a similar trend, with a maximum value of 0.14 at 70°C (Figure 5e; Figure S28) At this temperature, the PBG overlaps with the fluorescence emission peaks to the greatest extent, resulting in maximum g_lum_ for L‐CPL. As the temperature further increased, chiral transfer became the dominant mechanism, R‐CPL increased gradually. Finally, the intensity of R‐CPL surpassed that of L‐CPL at 100°C, with a maximum g_lum_ for R‐CPL (Figure S28). This trend aligns with the proposed competition between PBG effects and chiral transfer effects, providing compelling evidence for the underlying mechanisms governing the CPL emission in these films.

In contrast to the CPL behavior, the CPRTP spectra of the films consistently exhibited negative signals, indicating R‐CPRTP emission (Figure 5d). This phenomenon is primarily attributed to the dominant role of chiral transfer in the governing of the CPRTP emission. As the drying temperature increased, both the CPRTP intensity and the g_lum_ displayed a non‐monotonic trend, initially increasing and then decreasing (Figure 5e: Figure S29). The maximum CPRTP emission intensity was observed at 90°C, with a corresponding g_lum_ value of −0.17. This is because phosphorescence emission coincides with the CD bands of the film to the greatest extent, resulting in a strong CPRTP signal.

**FIGURE 6:**
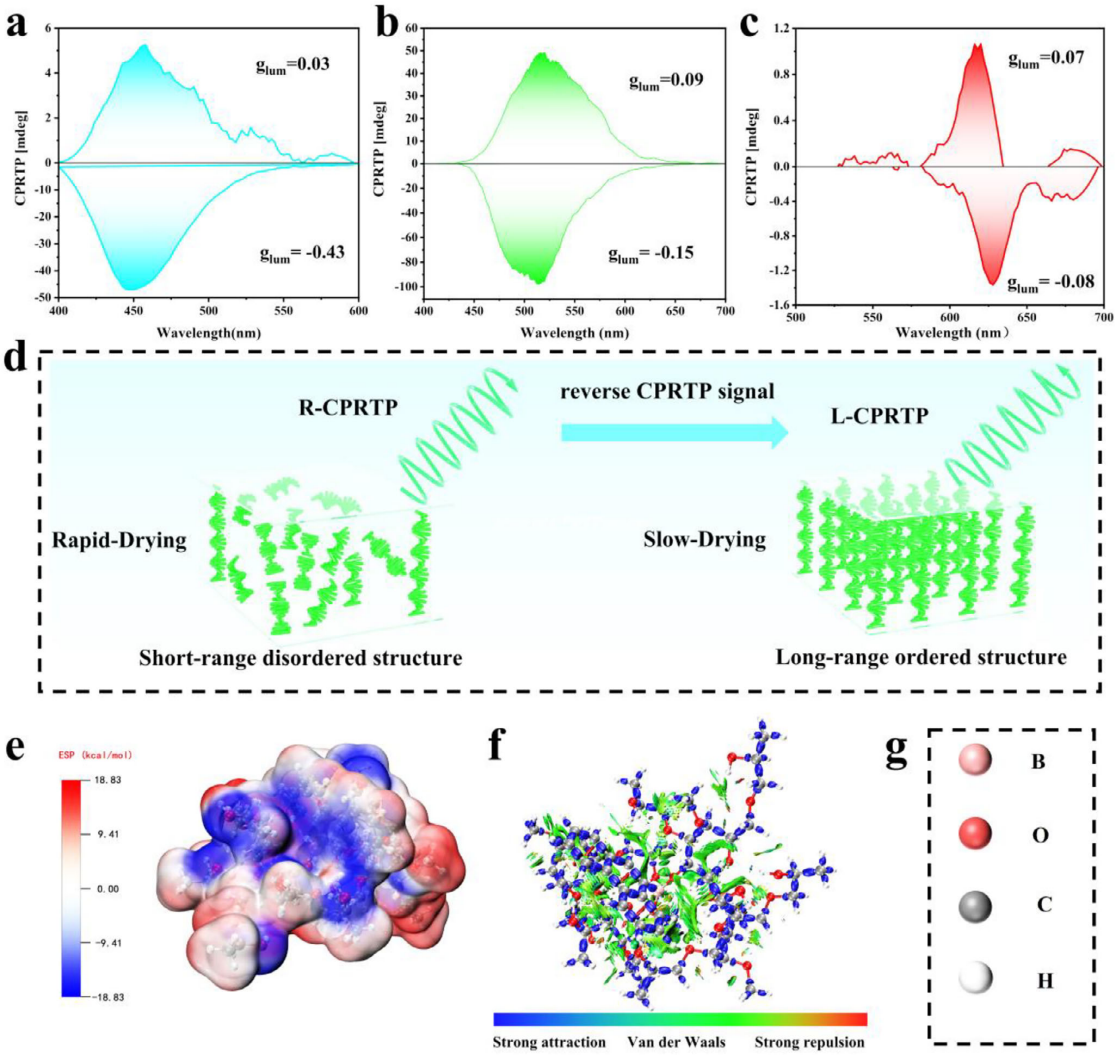
(a) CPRTP spectra of thin and thick g‐3‐bip at 80°C. (b) CPRTP spectra of thin and thick g‐9‐phe at 90°C. (c) CPRTP spectra of thin and thick g‐1‐py at 100°C. (d) Schematic diagram of the helical structure of HPC during slow drying and rapid drying. by controlling the drying kinetics. (e) ESP distribution of RTP‐HPC. (f,g) IGMH analysis of the intermolecular interactions of RTP‐HPC.

To further demonstrate the effect of drying kinetics on CPL and CPRTP, a series of HPC films with varied thicknesses were dried at constant temperature (80°C). The intensity of the CD peaks (Figure S30) increased progressively with film thickness, which is caused by an increase in the degree of long‐range ordering of the film (increased thickness, slower drying rate) (Figure 6d). The CPL spectra of these films revealed thickness‐dependent CPL behaviors (Figure S31a). Thinner films exhibited both positive and negative CPL signals, whereas thicker films displayed exclusively positive signals. We hypothesize that the short‐range disordered structure in thinner films allows for the coexistence of CPL emission governed by both PBG effects and chiral transfer mechanisms. In contrast, in thicker films, more long‐range ordered structure formed, enhancing the dominance of PBG effects in CPL generation. This hypothesis was corroborated by the g_lum_ variation curves (Figure S31b). Similarly, the CPRTP also show thickness‐dependent behaviors. Thinner films produced negative CPRTP signals before 0.7 mm, with corresponding negative g_lum_ values (Figure S32). While for the films thicker than 0.7 mm, the CPRTP signal inverted, generating positive CPRTP emission. Concurrently, the g_lum_ curves transitioned to positive values, further verifying the interplay between chiral transfer and PBG effects.

Based on the drying kinetics effect on their chiroptical properties, HPC films with opposite handed CPRTP and different afterglow colors were prepared. To achieve maximum g_lum_ for CPRTP, thin and thick films of g‐3‐bip, g‐9‐phe, and g‐1‐py were prepared with overlap their phosphorescence emission bands with PBG. As evidenced by the CPRTP spectra (Figure 6a–c), the thinner films generated a negative CPRTP signal, while the thicker counterparts exhibited a signal inversion, producing a positive CPRTP signal. The g_lum_ directly correlated with the sign of the CPRTP signal. Notably, the thin film of g‐3‐bip displayed the largest negative g_lum_ value of −0.43, followed by g‐9‐phe (−0.15) and g‐1‐py (−0.08). Conversely, the thick film of g‐9‐phe achieved the highest positive g_lum_ value (0.09), with g‐3‐bip (0.03) and g‐1‐py (0.07) showing progressively weaker responses. The parallel trend between g_lum_ values and CPRTP signal polarity conclusively validates the proposed mechanism (Figure S33).

The observed chiral transfer in RTP‐HPC films is likely mediated by interactions between arylboronic acid and HPC. To elucidate the interactions between HPC and arylboronic acids, DFT calculations were performed using 9‐phenanthrenylboronic acid as a representative model. ESP analysis revealed complementary positive and negative electrostatic regions between the RTP‐HPC molecules, indicative of hydrogen bonding interactions (Figure 6e). Furthermore, IGMH was employed to visualize noncovalent interactions between arylboronic acid and HPC. As shown in Figure 6f,g, distinct hydrogen bonds (blue isosurfaces) and pronounced van der Waals interactions (green isosurfaces) are observed, confirming strong intermolecular association between arylboronic acids and the HPC matrix. These strong interactions establish sufficient conditions for efficient chiral transfer [71]. As a result, L‐CPRTP with different PBGs and R‐CPRTP induced by intermolecular interactions coexists in RTP‐HPC. Meanwhile, compared with existing bio‐based and photoactivated CPRTP systems, RTP‐HPC also exhibits multi‐color, long‐life, and high‐glum, which expands its applications in real‐world scenarios (Table S1).

Furthermore, the mechanical properties of RTP‐HPC films were enhanced following the incorporation of PEG, in comparison to the films devoid of PEG (Figures S34 and S35). The FTIR spectra (Figure S36) revealed that the addition of PEG induces a narrowing and redshift of the hydroxyl stretching vibrational band (3300–3500 cm^−^^1^), indicating the formation of hydrogen bonds between HPC and PEG molecules. These increased hydrogen bonds ultimately result in a significant improvement in the mechanical properties of the HPC film, which expands their real‐life applications.

### 2.5 Humidity‐Responsive CPRTP

**FIGURE 7:**
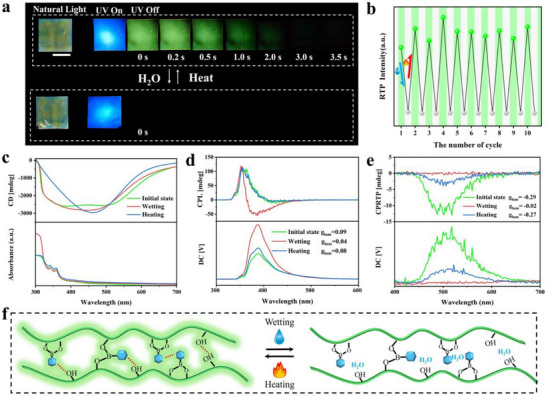
Water/heating stimuli‐responsive CPRTP property. (a) Photographs of the reversible water/heating fumigation processes for g‐9‐phe (after 30 s of irradiation from UV light), scale bar = 1 cm. (b) Stability of g‐9‐Phe RTP's water/heat stimulus response over 10 cycles. (c) CD spectra; (d) CPL spectra; (e) CPRTP spectra of the reversible water/heating fumigation processes for g‐9‐phe. (f) Schematic illustration of water/heat stimulus‐responsive mechanism.

Given the susceptibility of phosphorescence emission to water, we systematically investigated the humidity‐responsive CPRTP properties of RTP‐HPC. Taking g‐9‐phe for example, upon fumigation at 98% relative humidity for 30 min, the film exhibited a pronounced phosphorescence burst (Figure 7a). Remarkably, the photo‐activated RTP of the composite film was fully restored after thermal treatment at 120°C for 20 mins. The g‐9‐phe demonstrated exceptional water/heat‐stimulated reversible response characteristics, with no significant degradation in phosphorescence properties observed over 10 consecutive cycles (Figure 7b). Next, we explored the humidity‐stimulated responsiveness of g‐9‐phe in the context of photo‐activated CPRTP. Notably, fumigation did not induce any significant shift in the peak position of CD spectrum of g‐9‐phe (Figure 7c). This observation indicates that the helical pitch and PBG of g‐9‐phe remained largely unaffected, thereby preserving the emission of CPL and CPRTP (Figure 7d,e; Figures S37 and S38). Specifically, the CPL emission of g‐9‐phe remained unchanged before and after fumigation, as both its fluorescence emission and PBG were impervious to water. However, since the phosphorescence emission of g‐9‐phe was quenched by water, its CPRTP emission was nearly eliminated after fumigation but recovered upon subsequent heating. Moreover, the sample maintained CPRTP emission throughout 10 water/heat stimulus response cycles, with no significant reduction in its g_lum_ or lifetime, demonstrating excellent cycling performance and long‐term stability (Figure S39).

To elucidate the underlying mechanism of the humidity‐responsive CPRTP properties, FTIR spectroscopy of g‐9‐phe was conducted (Figure S40). The spectra revealed a distinct peak at approximately 3400 cm^−^^1^, attributable to water and hydroxyl groups associated with neighboring g‐9‐phe molecules. Following water fumigation, the intensity of this peak increased significantly, and its shape broadened compared with the heat treated HPC film, confirming a substantial increase in water content within the film [18]. Additionally, we treated g‐9‐phe with various organic solvents, including ethanol, acetonitrile, and dimethyl sulfoxide. While these treatments led to a reduction in phosphorescence intensity, the phosphorescence emission persisted. In contrast, water completely quenched the phosphorescence (Figure S41). Based on these findings, we propose that the ingress of water molecules into g‐9‐phe disrupts the hydrogen bonding network between adjacent g‐9‐phe molecules, thereby destabilizing the rigid environment necessary for RTP upon thermal treatment, the reformation of intermolecular hydrogen bonds within the cellulose matrix restores the RTP emission (Figure 7f).

### 2.6 Potential Applications for RTP‐HPC

**FIGURE 8:**
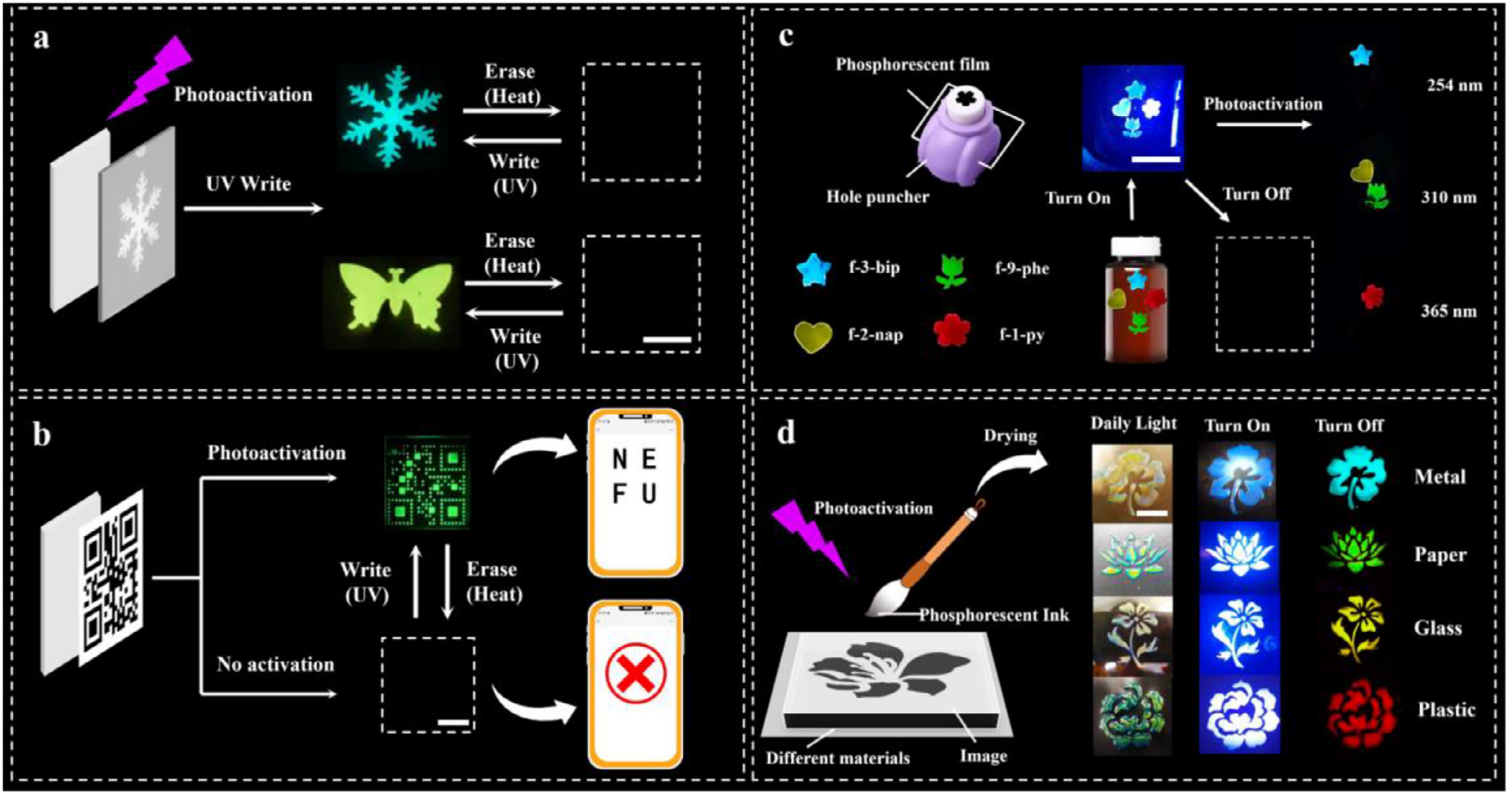
Potential applications for RTP‐HPC. The schematic illustration of the application process for (a), and (b) anti‐counterfeit, b) phosphorescent decorative, and (c) screen printing as phosphorescent ink, scale bar = 1 cm.

Leveraging its multi‐stimulus responsive CPRTP properties and moldable nature, RTP‐HPC have been successfully implemented in photo‐programable information storage, phosphorescent decoration, and afterglow ink applications. First, a rewritable smart film was fabricated based on the photo‐responsive afterglow of RTP‐HPC, enabling programmable and reusable lifetime‐encrypted security tags through inkless mask photolithography. The photoactivated afterglow patterns were generated on different substrates using inkless UV mask lithography (Figure 8a). Further, we also used this technique to write a QR code pattern on f‐9‐phe, which can be scanned with a cell phone to read “NEFU” after continuous excitation of the light source. The unactivated f‐9‐phe showed the opposite phenomenon (Video S3). The QR code was also erased after heating at 50°C for 1 min (Figure 8b). Thus, by combining the photoactivation and thermal deactivation of afterglow emission, reversible write‐read‐erase cycles for displaying snowflakes, butterflies, and QR codes with different afterglow patterns at high resolution can be easily achieved in these RTP‐HPC films (Figure S42). Based on its easy processing characteristics, RTP‐HPC films were further evaluated as multi‐color photoactivated phosphorescent decorative elements. Using an embossing machine, we imprinted distinct patterns onto the films, which were then applied to medicine bottle surfaces. Under excitation, all four patterns exhibited similar fluorescence; however, upon cessation of the light source, each film displayed clearly distinguishable phosphorescent emissions‐blue (254 nm), green (310 nm), yellow (310 nm), and red (365 nm) (Figure 8c). These embossed patterns underscore the material's potential for advanced decorative applications with dynamic visual effects. Furthermore, RTP‐HPC was formulated into a solid ink and successfully employed in screen‐printing various patterns on different substrates. As illustrated in the accompanying figure, the printed features exhibit vibrant structural colors under ambient light, uniform fluorescence upon excitation, and diverse afterglow emissions when the light source is removed (Figure 8d; Figure S43). Unlike solvent‐based systems that restrict substrate compatibility, RTP‐HPC enables direct printing on numerous surfaces—including metal, paper, glass, and plastic—significantly broadening its application scope. As a biomass‐derived phosphorescent material, RTP‐HPC demonstrates outstanding biodegradability. When buried in soil, the films undergo complete decomposition within 8 days (Figure S44). This rapid degradation profile not only minimizes environmental impact but also enhances the material's appeal for sustainable, eco‐friendly applications.

## 3 Conclusion

In conclusion, we have successfully developed a novel class of photo‐activatable CPRTP from bio‐based HPC materials via B‐O covalent bonding strategy. These bio‐based systems feature multicolor emission, tunable chirality, significant enhanced lifetime up to 1.57 s, and high‐g_l__um_ of 0.43 after photo‐activation. The initially weak phosphorescence in RTP‐HPC arises from the limited confinement ability of HPC, which permits residual oxygen to quench the triplet state. Upon continuous UV irradiation, the consumption of trapped oxygen activates intense and persistent RTP. Importantly, HPC films exhibit tunable CPL and CPRTP by controlling the drying temperature. Meanwhile, owing to the competition effect of PBG and chirality transfer in the RTP‐HPC films with different thicknesses, both R‐CPRTP and L‐CPRTP with tunable afterglow colors can be achieved in HPC films. Notably, reversible on/off CPRTP with varied g_lum_ (−0.29–−0.02–−0.27) can be realized by water/heat treatment over multiple cycles. In addition, the rapid biodegradable CPRTP materials show promise applications in anti‐counterfeiting, phosphorescent decorations and eco‐friendly phosphorescent inks. This work provides guidance for designing programmable and high‐performance photo‐activated CPRTP materials from sustainable bioresources, expanding their large‐scale production and advanced applications.

## Conflicts of Interest

The authors declare no conflicts of interest.

## Supporting information




**Supporting File 1**: advs73778‐sup‐0001‐SuppMat.docx.

**Supporting File 2**: advs73778‐sup‐0002‐MovieS1.mp4.

**Supporting File 3**: advs73778‐sup‐0003‐MovieS2.mp4.

**Supporting File 4**: advs73778‐sup‐0004‐MovieS3.mp4.

## Data Availability

The data that support the findings of this study are available from the corresponding author upon reasonable request.
