# Supplementary material for: Achieving Photo‐Activated Circularly Polarized Room Temperature Phosphorescence from Natural Biopolymers
Source: Adv Sci (Weinh). 2026 Jan 11;13(10):e23073. doi: 10.1002/advs.202523073 (PMC12915112; doi:10.1002/advs.202523073)
Supplement: Supplementary file 1 — Supporting File 1: advs73778‐sup‐0001‐SuppMat.docx. [file ADVS-13-e23073-s004.docx]

**Supporting Information**

Achieving Photo-activated Circularly Polarized Room Temperature Phosphorescence from Natural Biopolymers

*Shaoyi Cao^1^, Mingcong Xu^1,2^*, Bang An^1^, Baoqi Li^2^, Wenye Sun^2^, Wenbo Cui**^2^, Rui Teng^2^, Chunhui Ma^1^, Sha Luo^1^, Bing Tian^1^, Zhijun Chen^2^, Shouxin Liu^1,2*^, and Wei Li^1,2*^*

1. State Key Laboratory of Woody Oil Resources Utilization, Northeast Forestry University, Harbin 150040, China

2. Key Laboratory of Bio-based Material Science and Technology of Ministry of Education, Northeast Forestry University, Harbin 150040, China

**KEYWORS** hydroxypropyl cellulose, photo-activation, circularly polarized room temperature phosphorescence, multi-color, chirality tunable

**Content**

[**Experimental Section** 3](#_Toc216266630)

[**Supporting Figures** 6](#_Toc216266631)

[**Supporting Table** 23](#_Toc216266632)

[**Reference** 24](#_Toc216266633)

# **Experimental Section**

***Materials:*** HPC (hydroxypropyl cellulose, 3-6 mPa·s) was purchased from Tichy Chemical Industrial Development Co., LTD. (Shanghai); 3-biphenylboric acid (purity: 98%), 2-naphthylboric acid (purity: 97%), 9-phenanthrene boronic acid (purity: 97%) and 1-binylboronic acid (purity: 95%), 2,2,6,6-Tetramethylpiperidine (TEMP) were purchased from Aladdin (Shanghai). All commercial reagents were purchased and used as received.

***Preparation of RTP-HPC powders:*** HPC suspension (10 g) was prepared in deionized water (500 mL). 3-biphenylboronic acid/2-naphthylbornic acid/9-phenanthracenylboronic acid/1-pyrenylboronic acid (50 mg) and ammonium hydroxide (10 mL) were added to the HPC suspension and heated at 80 °C for 20 min. Subsequently, the obtained suspensions were poured into centrifuge tubes and freeze-dried to obtain RTP powder. These powders were successively named p-3-bip, p-2-nap, p-9-phe and p-1-py.

***Preparation of RTP-HPC films.*** RTP powder was prepared as a solution with a mass fraction of 57 wt% using the following method. Appropriate amounts of RTP powder and deionized water were added to a beaker, with the powder being added to the water to ensure uniform mixing. Immediately after mixing the components in the mixing cup, the sample was subjected to centrifugation at 10,000 RPM for 10 minutes. The samples were then removed and allowed to stand for approximately 3 to 5 minutes, then mixed a second time at 10,000 RPM for 10 minutes. This procedure was repeated until the solution was free of bubbles. Subsequently, 0.5 milliliters of the solution was transferred into a mold (3 x 3 centimeters) using a syringe. The mold was then placed into an oven, where it was left to dry. This process was carried out until the water had fully evaporated, resulting in a film with a thickness of 0.2 millimeters, which were named f-3-bip, f-2-nap, f-9-phe and f-1-py.

***Preparation of PEG&RTP-HPC films.*** The preparation of the PEG&RTP-HPC films was carried out in accordance with the same protocol as the RTP films, with the sole distinction being that the desired amount of PEG was mixed with water prior to the addition of the RTP powder. The mass fraction of PEG relative to the RTP powder was 10 wt%, and the mass fraction of the RTP powder in solution was a constant 57 wt%. The remaining steps in the preparation protocol for the PEG&RTP films were identical to those for the RTP films, as were the remaining steps in the preparation scheme. Subsequently, RTP films with a thickness of approximately 0.3 mm were obtained, and they were named g-3-bip, g-2-nap, g-9-phe, and g-1-py.

***Measurements:*** UV-absorption spectra were collected using a Lambda1050+ UV-visible NIR spectrophotometer (PerkinElmer Inc., USA). Fourier transform infrared (FTIR) spectra were recorded in the range of 500-4000 cm^-1^ by a Nicolet iS10 FTIR spectrometer (Thermo Scientific, USA). Surface morphology was observed using a FEI Quanta 200 microscope (Thermo Fisher Scientific, Germany). XPS spectra were measured using a Thermo Scientific K-Alpha (Thermo Scientific, USA) spectrometer. Fluorescence and phosphorescence spectra were obtained using an Agilent Cary Eclipse fluorescence spectrophotometer. Single-linear state oxygen radical detection using EPR

(BrunkerA300, Germany). The XRD patterns were obtained by X-ray diffraction (Rigaku Ultima IV, Japan). The measurement of phosphorescence lifetimes was conducted using an Edinburgh FLS 1000 instrument. Subsequently, photographs and videos were captured under ambient conditions using an Honor X50 cell phone. The mechanical properties of RTP films were investigated using an electronic universal testing machine with a load cell of 50 and a test speed of 5 mm min^-1^. Circular dichroism spectra were recorded on a JASCO J-1500 CD spectrometer. The CPL and CPRTP spectra were measured using a JASCO CPL-300 spectrophotometer.

***Theoretical calculation***: All calculations were performed using both Gaussian 16 and ORCA 6.1.0 software packages^[1]^. Geometry optimizations were conducted with the B3LYP functional^[2]^, in combination with Grimme’s D3(BJ)^[3,4]^ dispersion correction, employing the 6-31G(d)^[5]^ and def2-SVP^[6]^ basis sets, respectively. The RIJCOSX approximation was applied to accelerate the computations in ORCA. Electronic excitation properties were evaluated using the M062X functional in Gaussian^[7]^, while single-point energy calculations for singlet states were carried out at the ma^[8]^-def2-TZVP level in ORCA. Wave function analyses were performed using Multiwfn (3.8 dev), including interaction region indicator (IRI)^[9]^ analysis for weak intermolecular interactions and quantitative mapping of electrostatic potential (ESP)^[10]^ extrema on van der Waals surfaces. Visualization of isosurfaces and molecular graphics were generated with VMD^[11]^ based on data exported from Multiwfn^[12]^.

# **Supporting Figures**

**
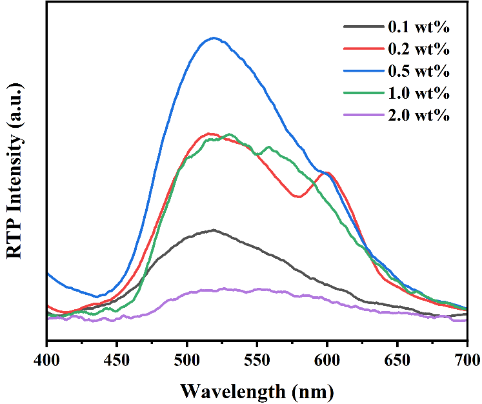
**

**Figure S1** Phosphorescence spectra of HPC with different doping concentration of 9-phenanthrene boronic acid.


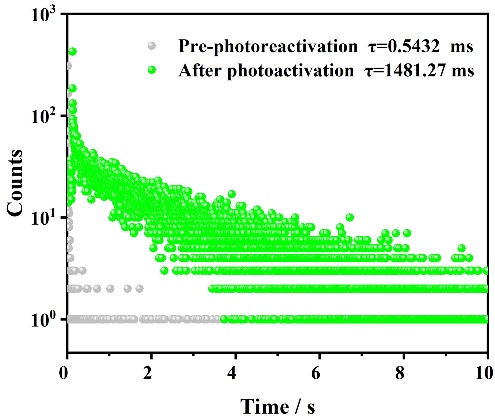


**Figure S2** Lifetimes of p-9-phe before and after photoactivation.


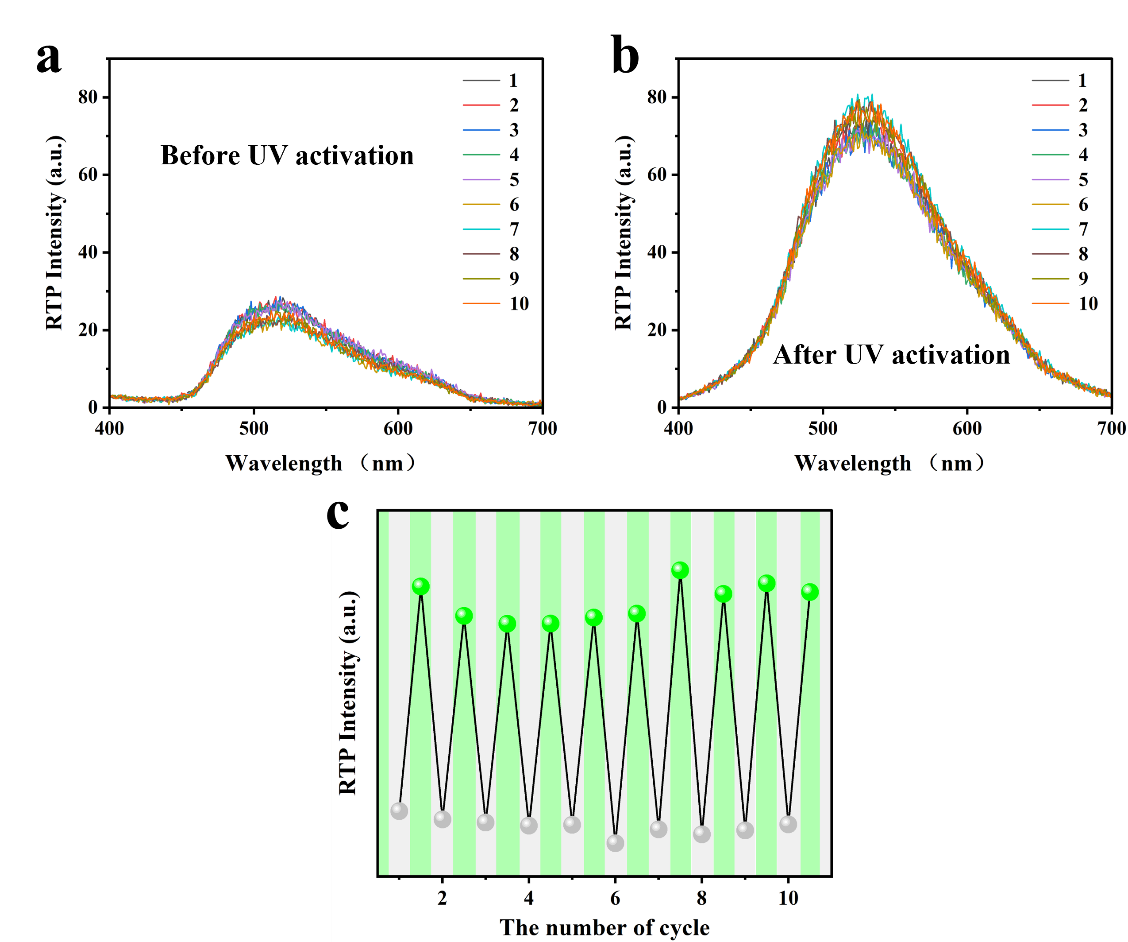


**Figure S3 a)** RTP spectra before photoactivation. **b)** RTP spectra after photoactivation. **c)** Schematic diagram of phosphorescence intensity cycling for p-9-phe after 10 photoactivation cycles.

**
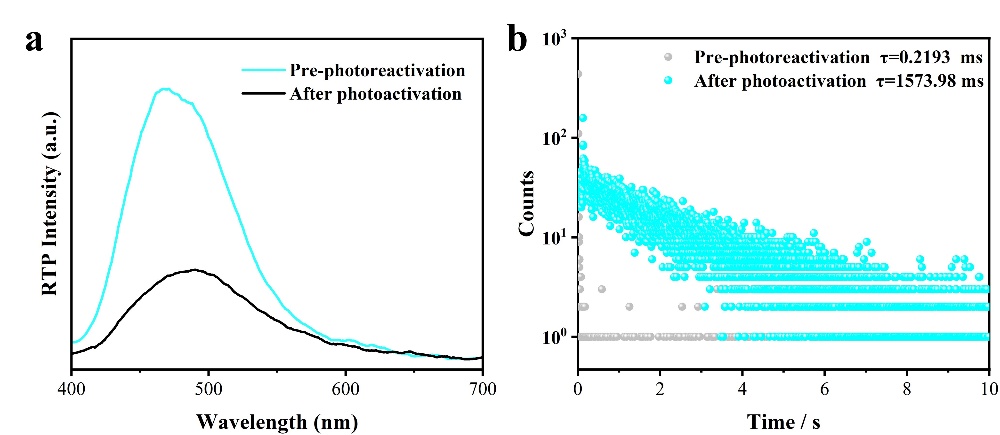
**

**Figure S4** The RTP spectra and lifetimes of p-3-bip before and after photoactivation.

**
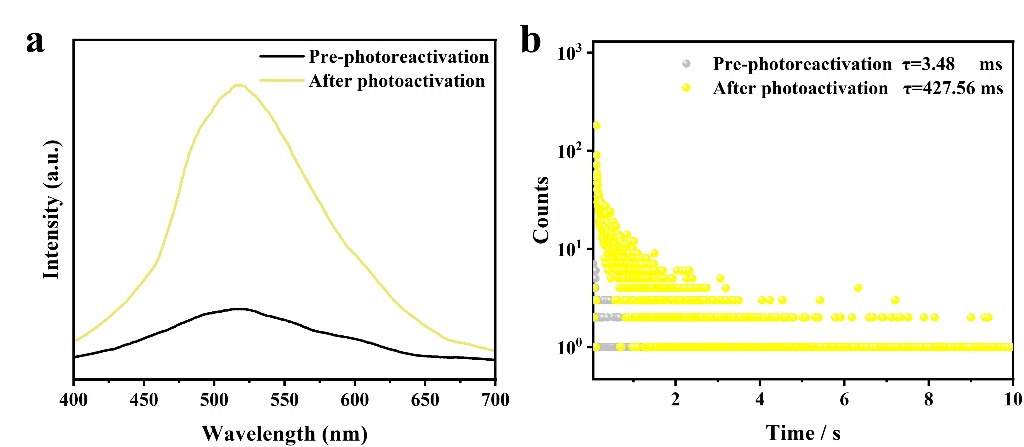
**

**Figure S5** The RTP spectra and lifetimes of p-2-nap before and after photoactivation.

**
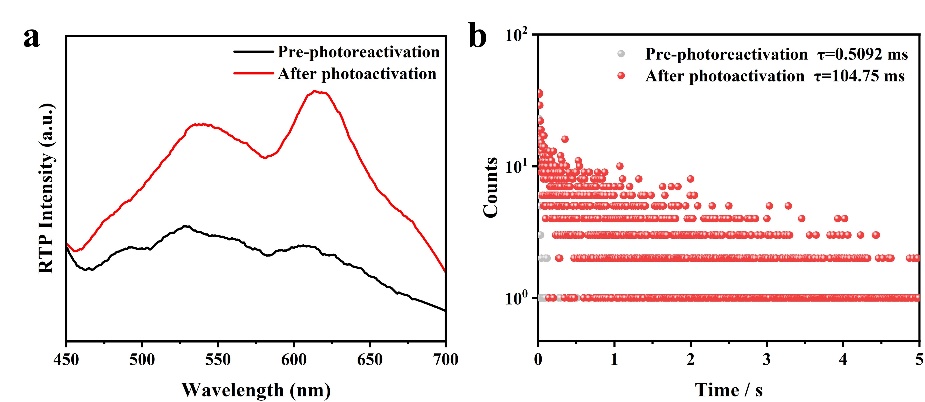
**

**Figure S6** The RTP spectra and lifetimes of p-1-py before and after photoactivation.


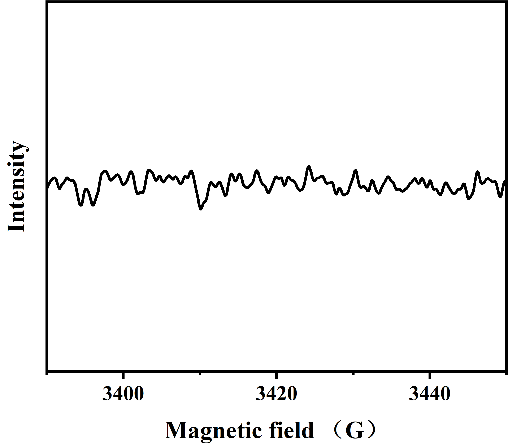


**Figure 7** EPR spectra of p-9-phe in N_2_.

**
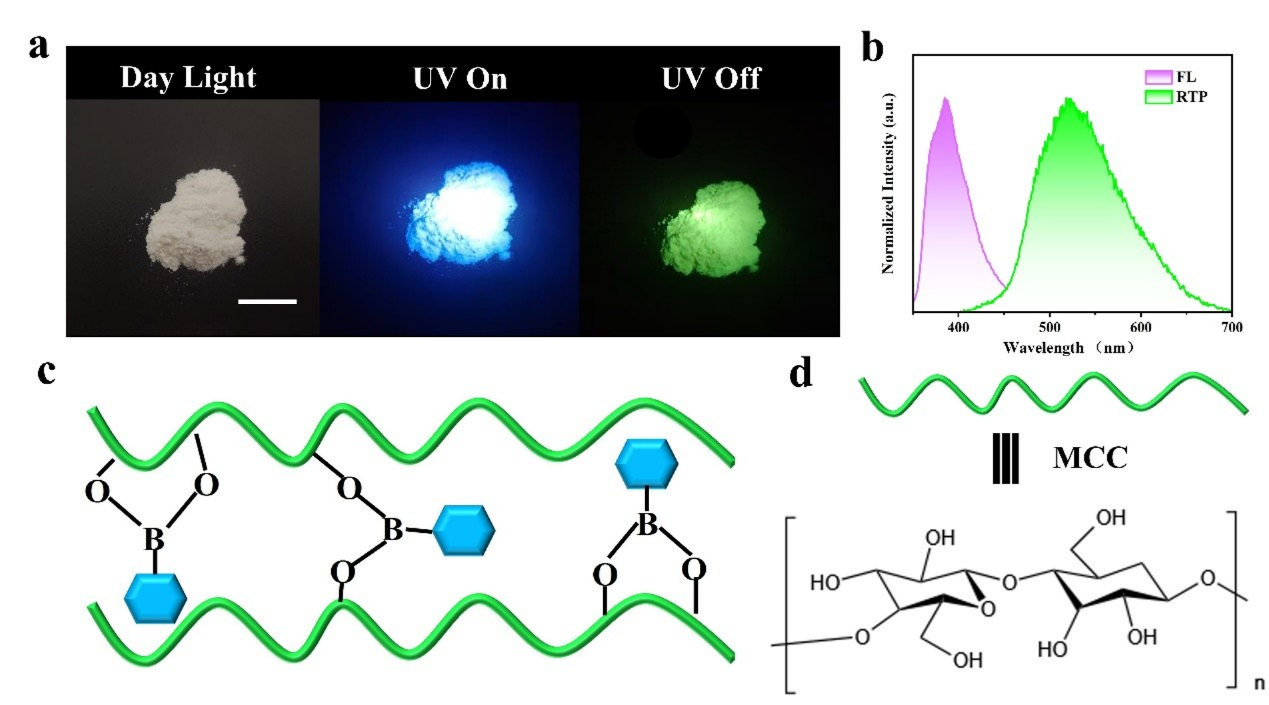
**

**Figure S8 a)** The image of MCC-RTP in day light, turn on and turn off; **b)** The FL and RTP spectra of MCC-RTP; **c, d)** The structural formula of RTP-MCC and MCC, scale bar = 1 cm.


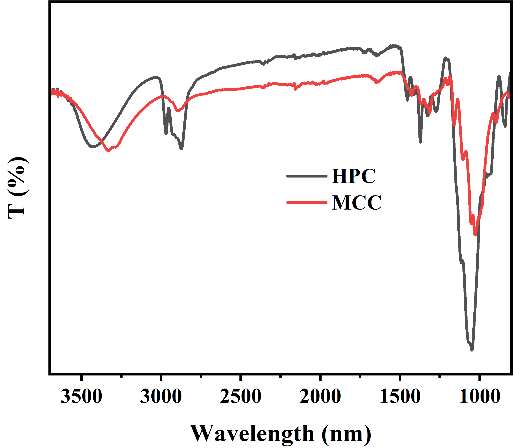


**Figure S9** FTIR spectroscopy of HPC and MCC.


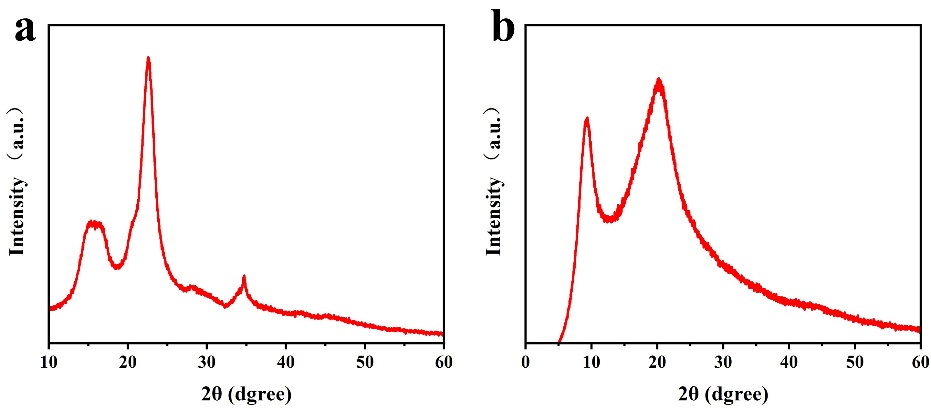


**Figure S10** XRD pattern of **a)** MCC, and **b)** HPC.


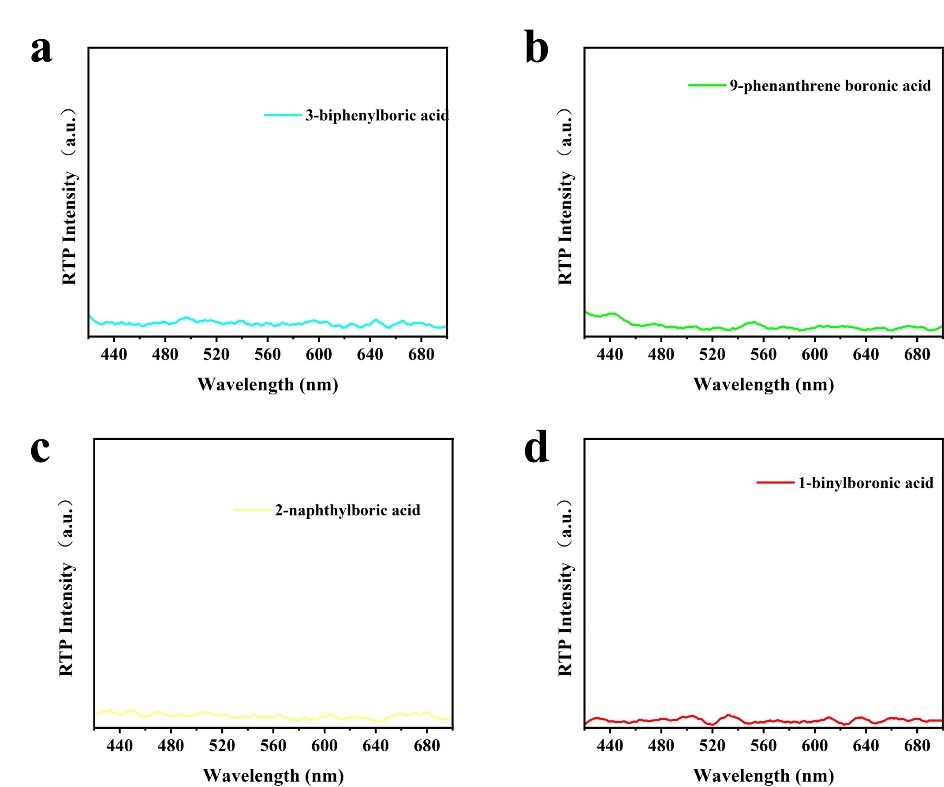


**Figure S11** The RTP spectra of **a)** 3-biphenylboric acid, **b)** 9-phenanthrene boronic acid, **c)** 2-naphthylbornic acid, and **d)** 1-binylboronic acid.

**
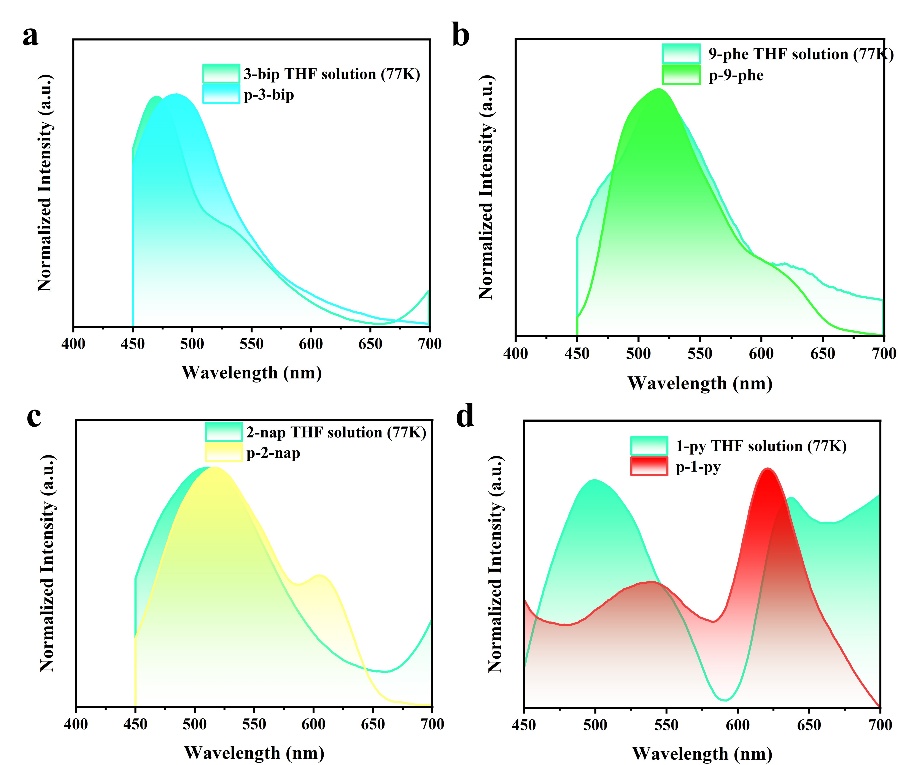
**

**Figure S12** Low temperature phosphorescence spectra and RTP spectra of **a)** 3-biphenylboric acid THF solution (10^-5^ M) and p-3-bip, **b)** 9-phenanthrene boronic acid THF solution (10^-5^ M) and p-9-phe, **c)** 2-naphthylboric acid THF solution (10^-5^ M) and p-2-nap, and **d)** 1-binylboronic acid THF solution (10^-5^ M) and p-1-py.

**
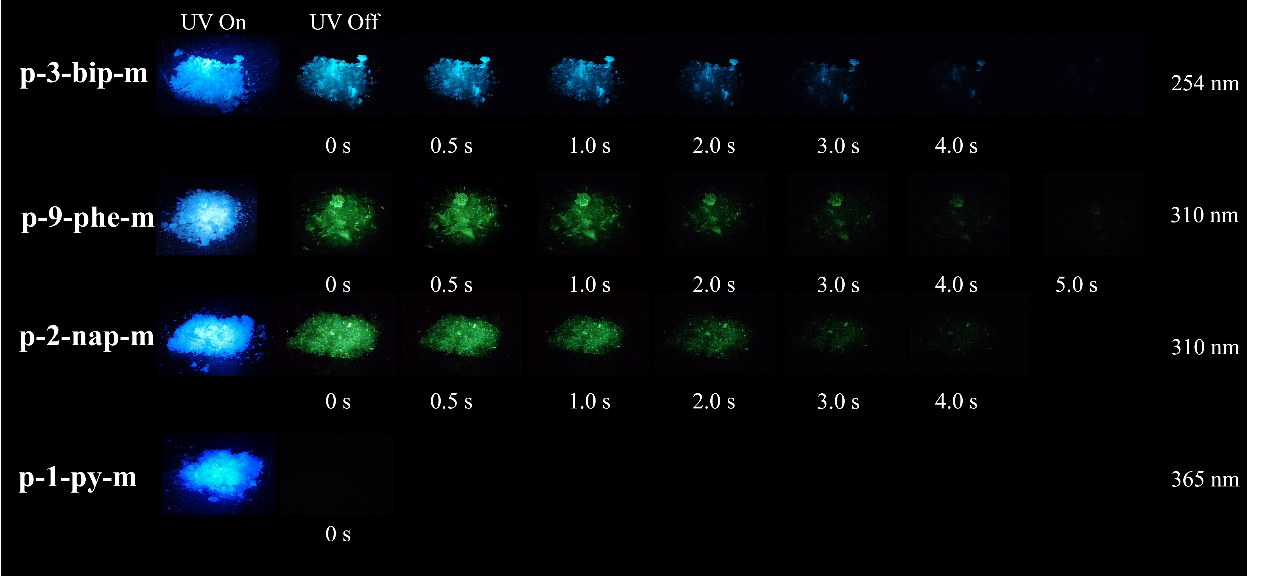
**

**Figure S13** Photos of p-3-bip-m, p-9-phe-m, p-2-nap-m, and p-1-py-m under different excitation lights.

**
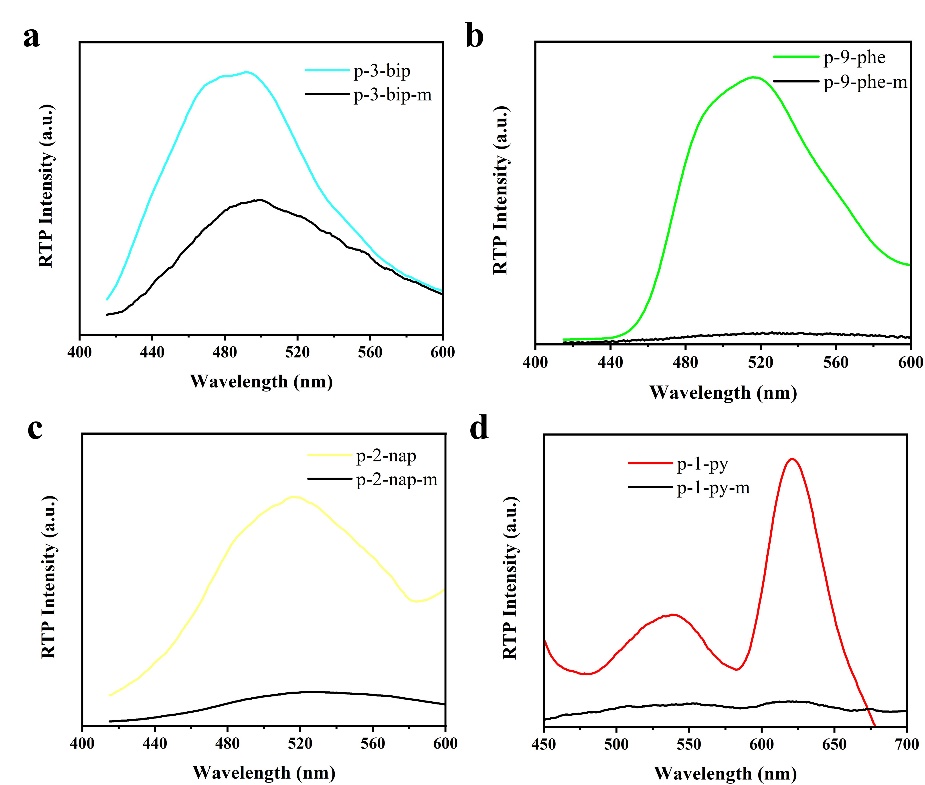
**

**Figure S14** RTP spectra of p-3-bip-m, p-9-phe-m, p-2-nap-m, and p-1-py-m under different excitation lights.

**
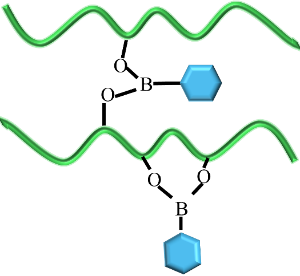
**

**Figure S15** Schematic illustration of the B-O covalent bonding between cellulose and arylboronic acids in RTP-HPC.

**
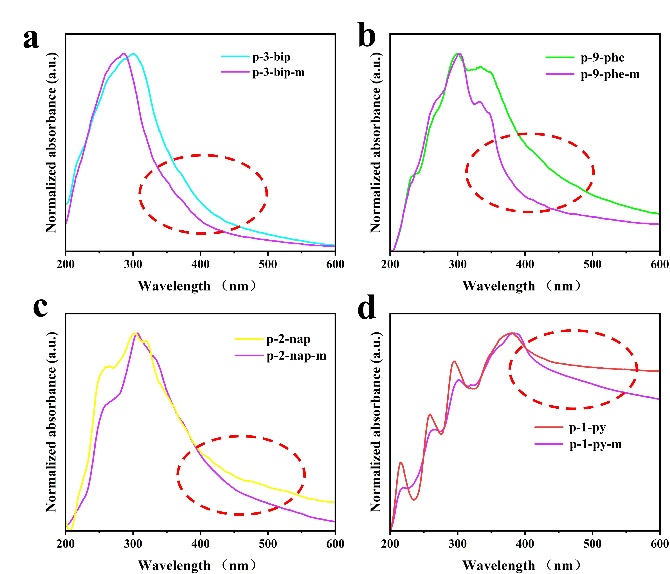
**

**Figure S16** Absorption spectra of p-3-bip, p-2-nap, p-9-phe, and p-1-py and their counterparts p-3-bip-m, p-2-nap-m, p-9-phe-m, and p-1-py-m.

**
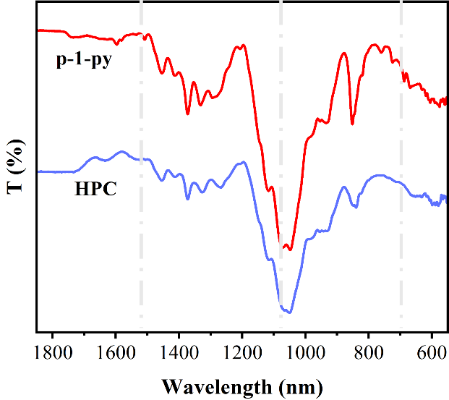
**

**Figure S17** FT-IR spectra of p-1-py and HPC.

**
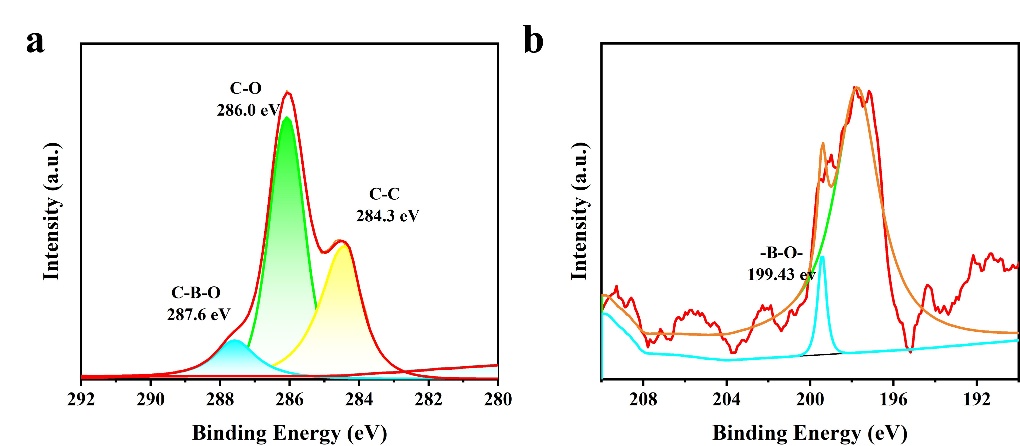
**

**Figure S18** High-resolution XPS spectra for **a)** C1s and **b)** B1s of p-1-py.

**
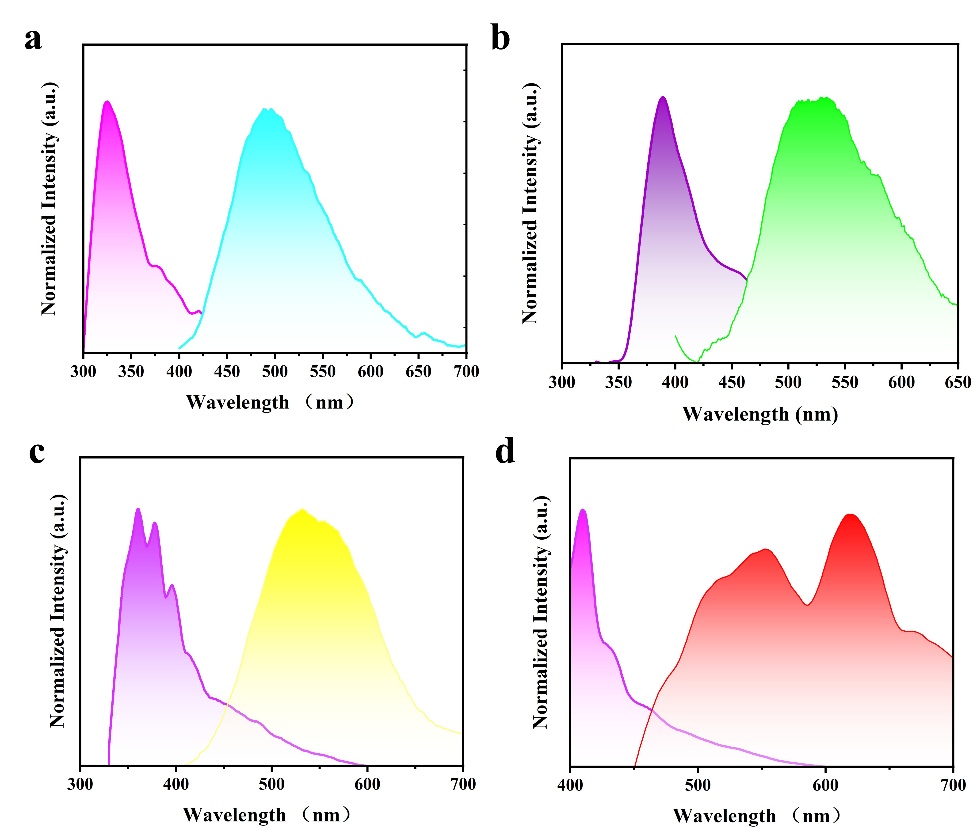
**

**Figure S19** Fluorescence and RTP spectra of f-3-bip, f-9-phe, f-2-nap, and f-1-py.

**
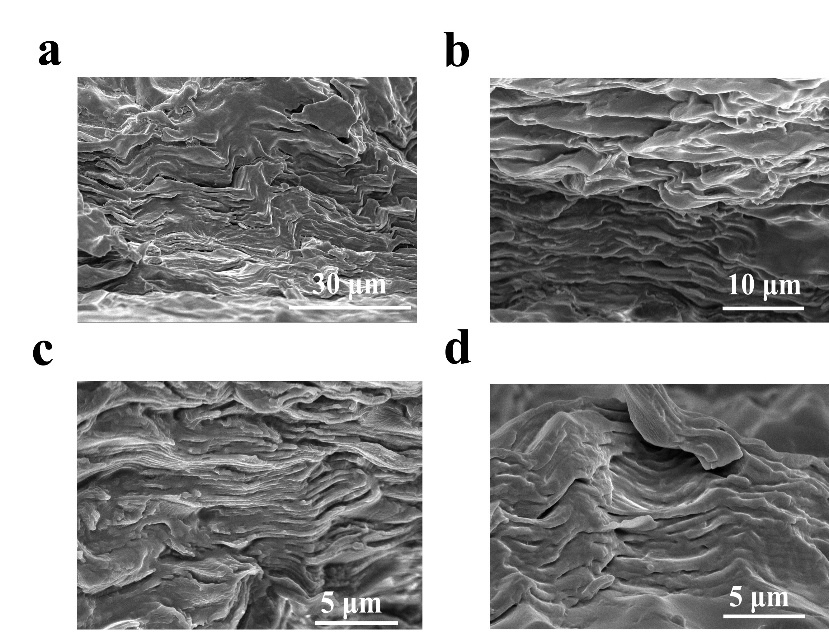
**

**Figure S20** SEM images of f-3-bip, f-9-phe, f-2-nap, and f-1-py.


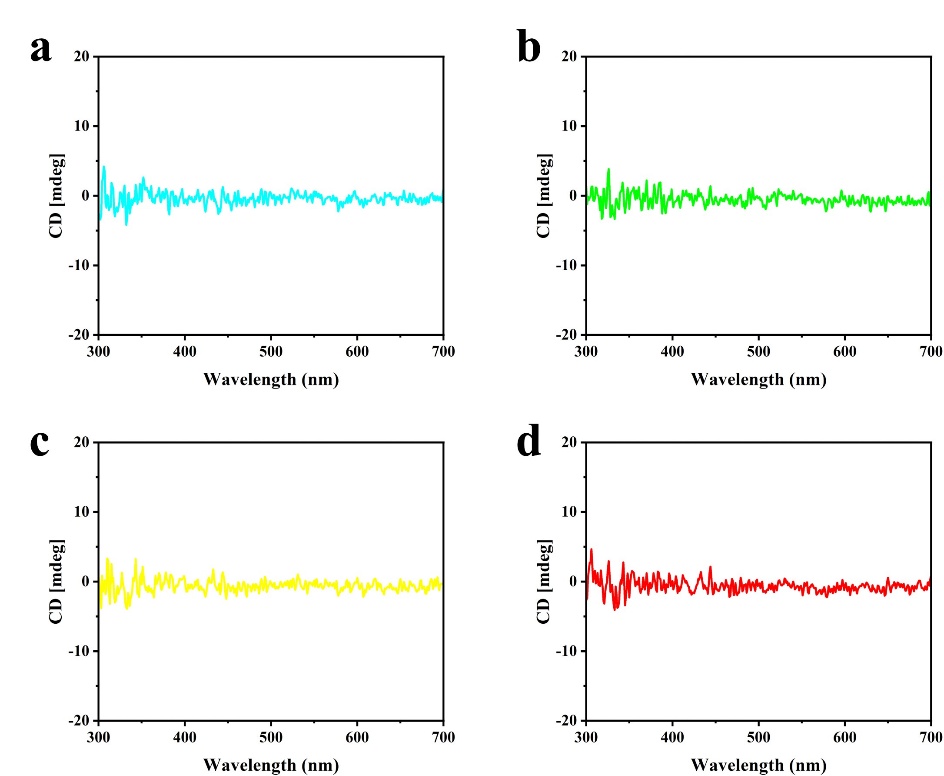


**Figure S21** CD spectra of **a)** 3-biphenylboric acid, b) 9-phenanthrene boronic acid, **c)** 2-naphthylboric acid, and **d)**1-binylboronic acid in THF.

**
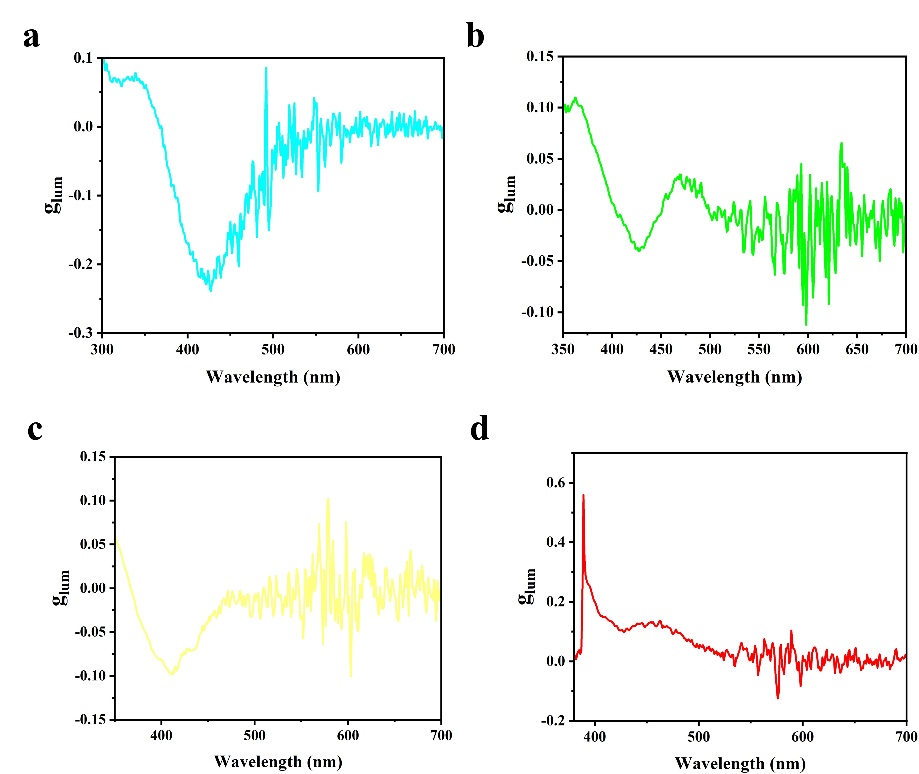
**

**Figure S22** g_lum_ curves of CPL of f-3-bip, f-9-phe, f-2-nap, and f-1-py.


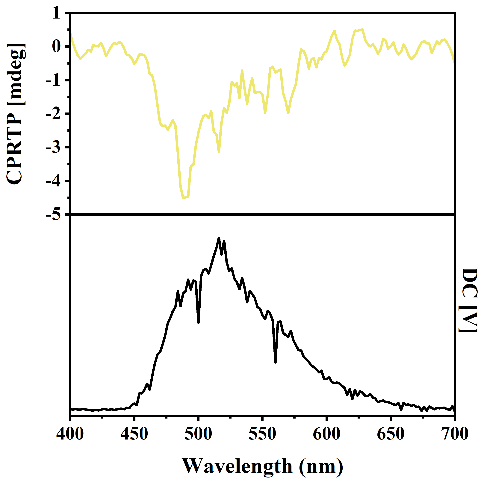


**Figure S23** The CPRTP spectra of f-2-nap.

**
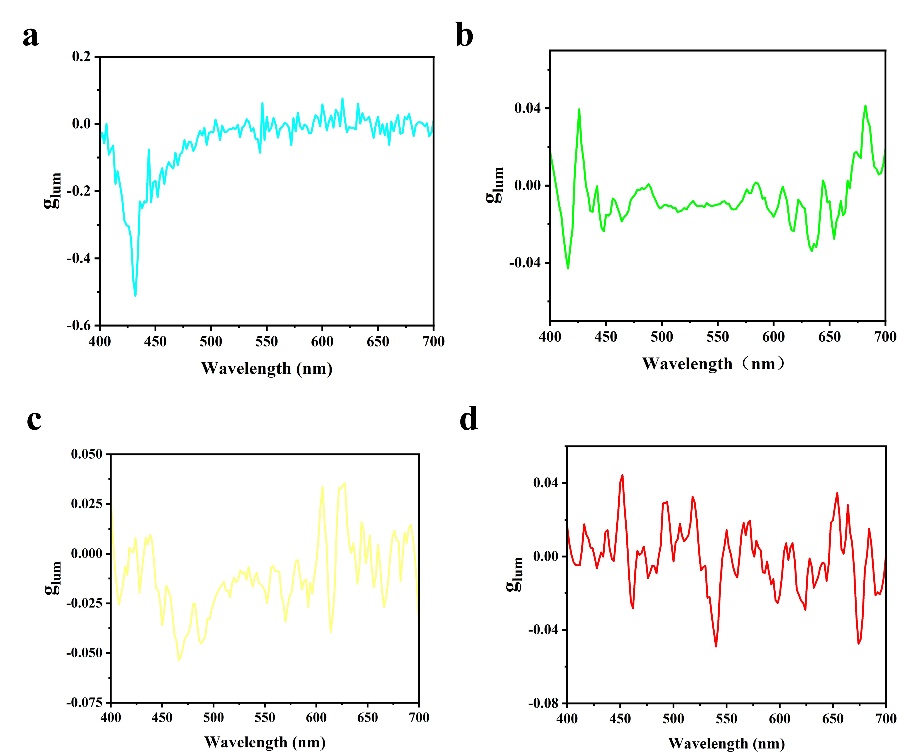
**

**Figure S24** g_lum_ curves of CPRTP of f-3-bip, f-9-phe, f-2-nap, and f-1-py.

**
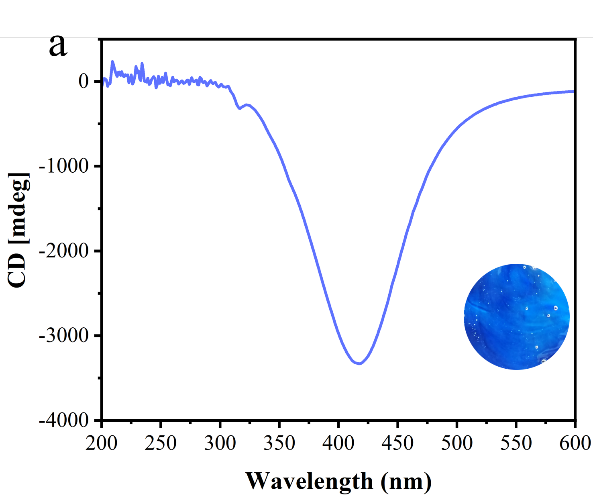
**

**Figure S25** CD spectra of pure HPC film without PEG.

**
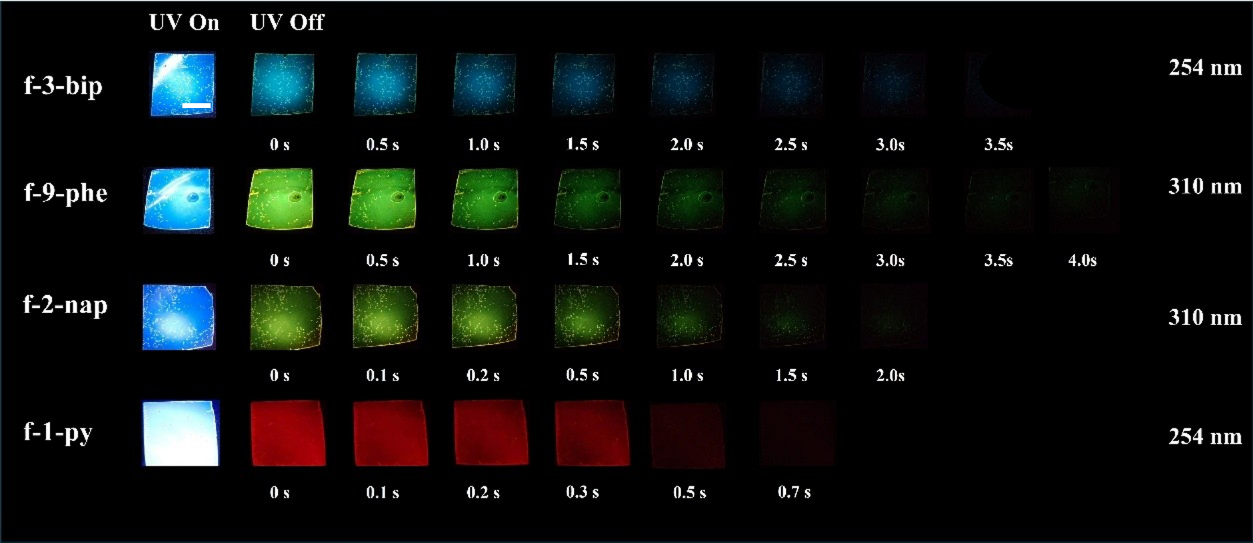
**

**Figure S26** Photos of f-3-bip, f-9-ph, f-2-na, and f-1-p under different excitation light, scale bar = 1 cm.

**
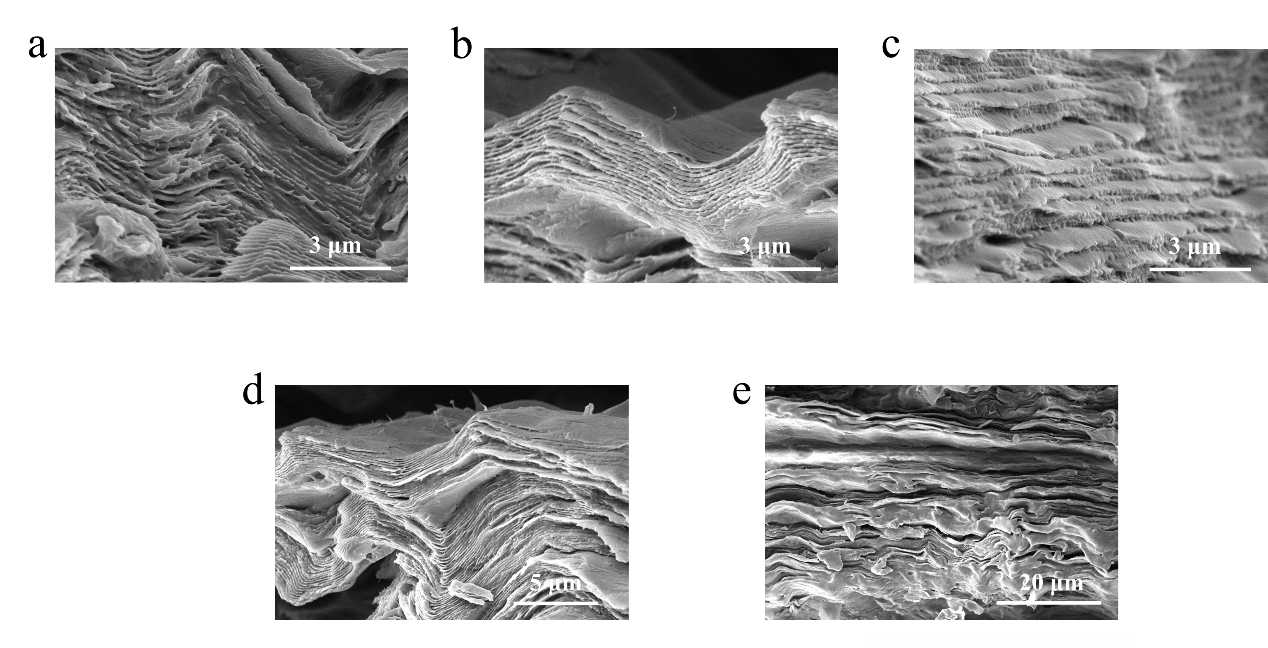
**

**Figure S27** SEM images of p-9-phe when dried at 60℃, 70℃, 80℃, 90℃, and 100℃.

**
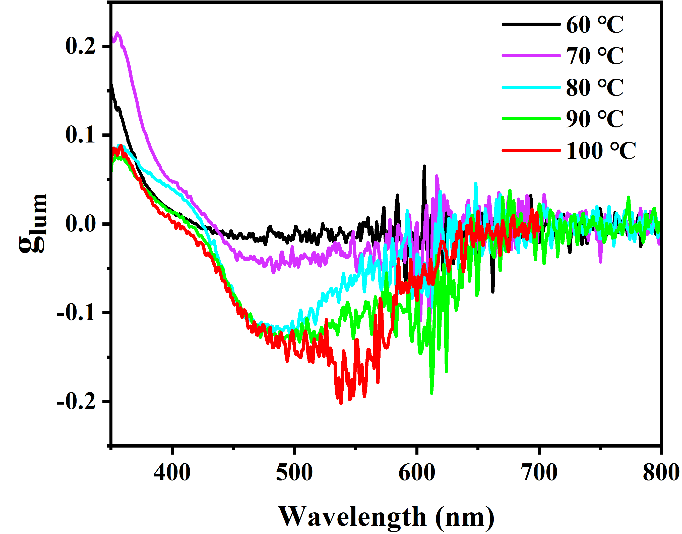
**

**Figure S28** g_lum_ curve of CPL of g-9-phe when dried at 60℃, 70℃, 80℃, 90℃, and 100℃.

**
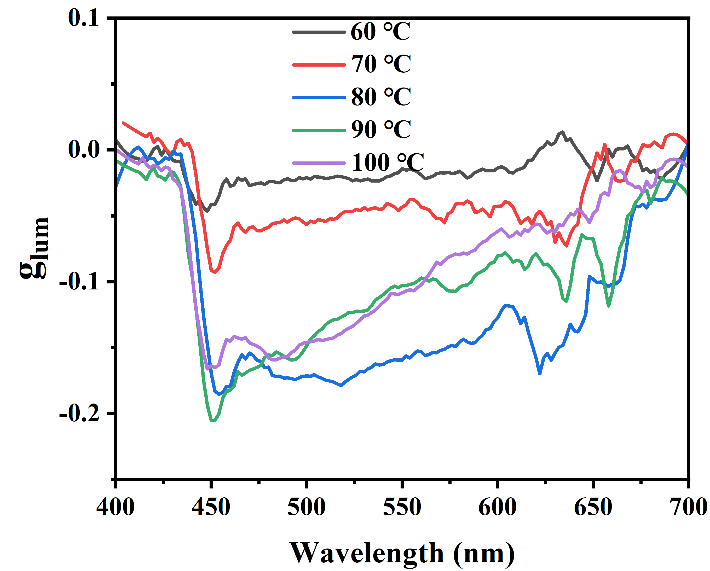
**

**Figure S29** g_lum_ curve of CPRTP of g-9-phe when dried at 60℃, 70℃, 80℃, 90℃, and 100℃.

**
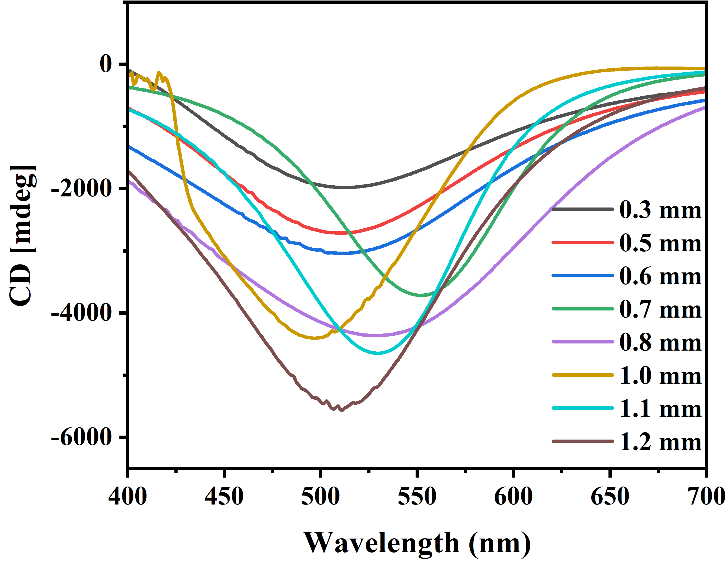
**

**Figure S****30** CD spectra of g-9-phe of different thicknesses.

**
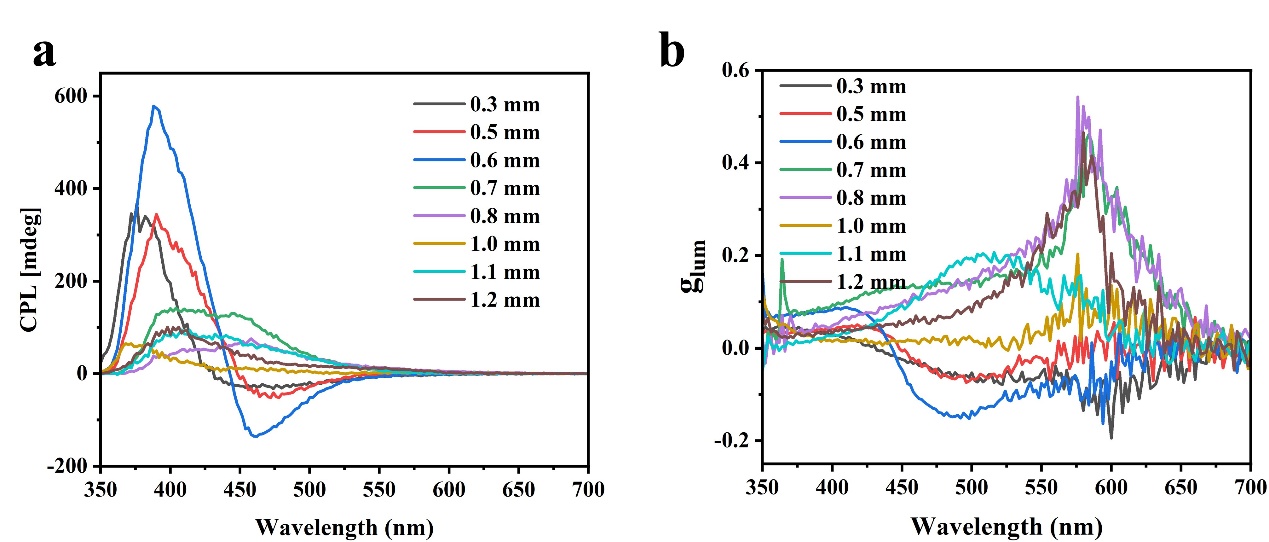
**

**Figure S31 a)** CPL spectra, and **b)** g_lum_ curve of g-9-phe of different thicknesses.

**
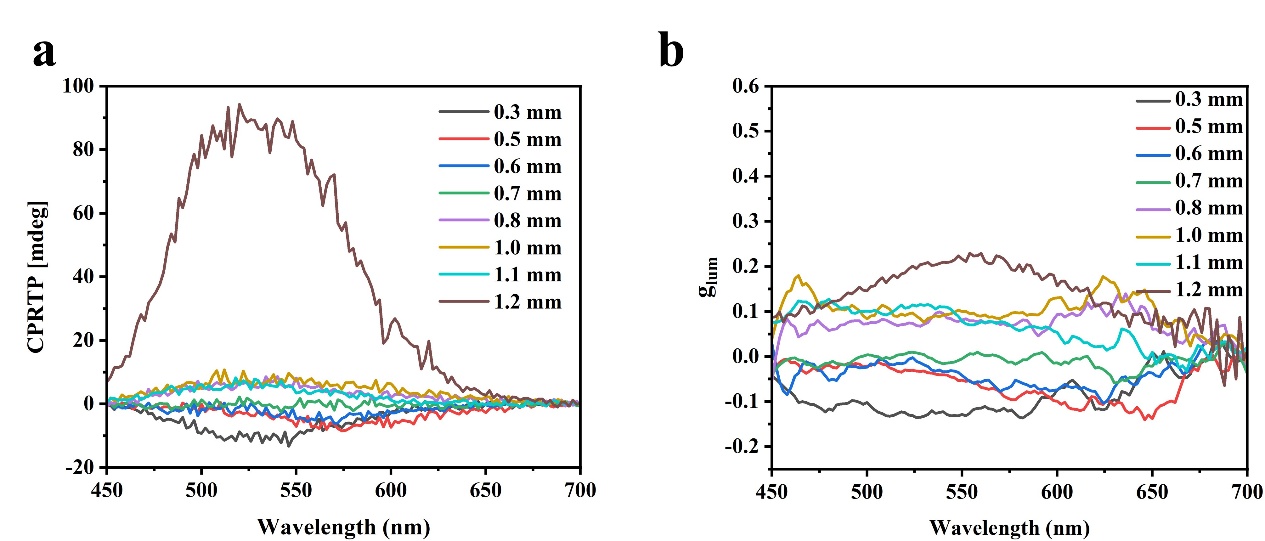
**

**Figure S32 a**) CPRTP spectra, and **b)** g_lum_ curve of g-9-phe of different thicknesses.

**
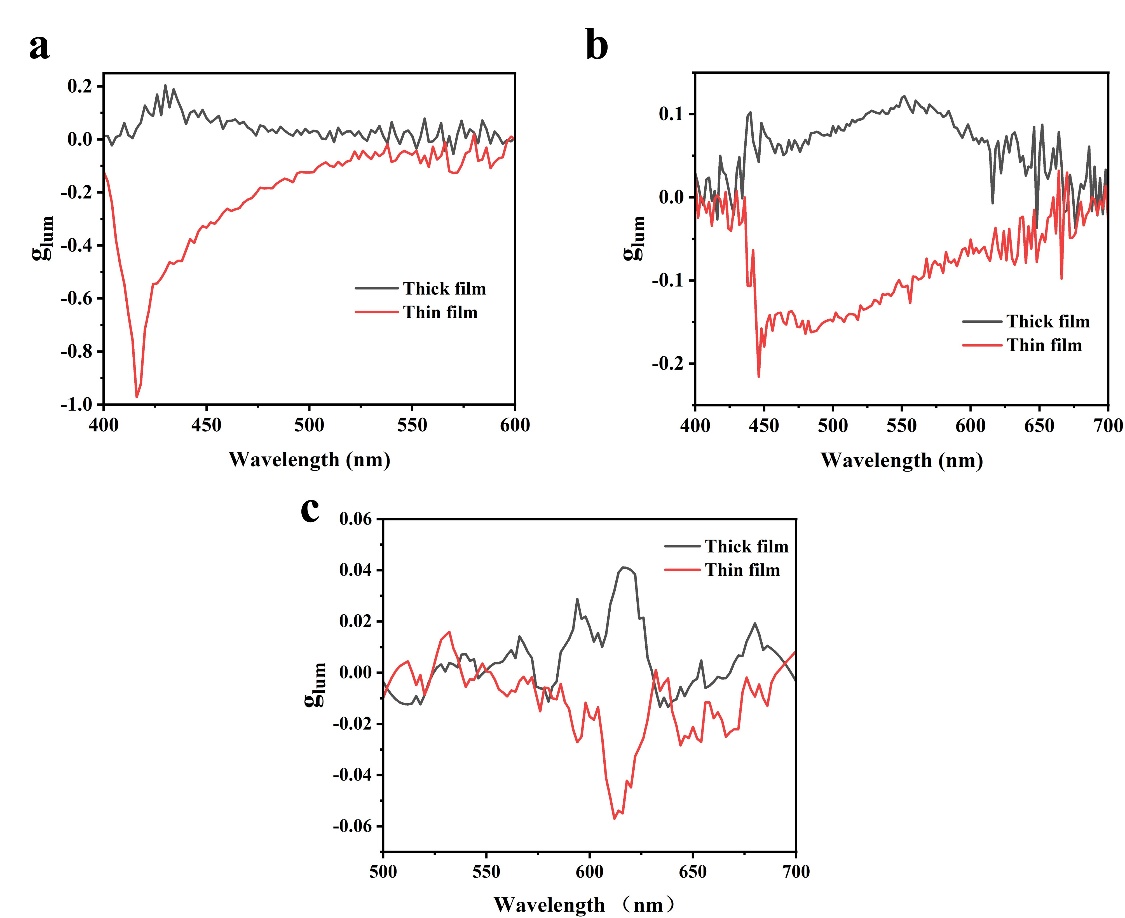
**

**Figure S33** g_lum_ curve of thin and thick **(a)** g-3-bip at 80 °C, **(b)** g-9-phe at 90 °C, **(c)**

g-1-py at 100 °C.

**
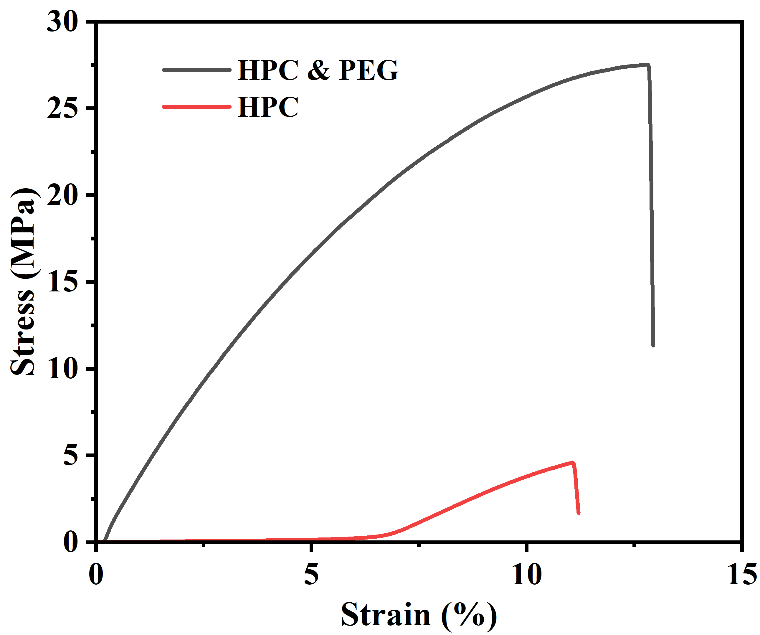
**

**Figure S34** Tensile–strain curves of pure HPC and HPC&PEG films.

**
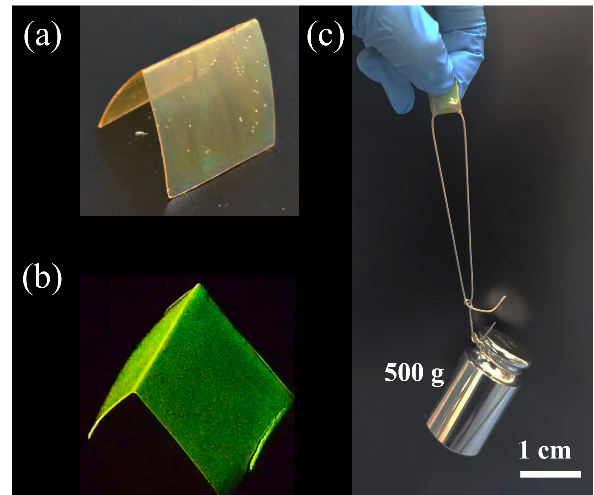
**

**Figure S35 a-b)** Photos of the bending of g-9-phe. **c)** Photograph of the g-9-phe during the lifting weight experiment.

**
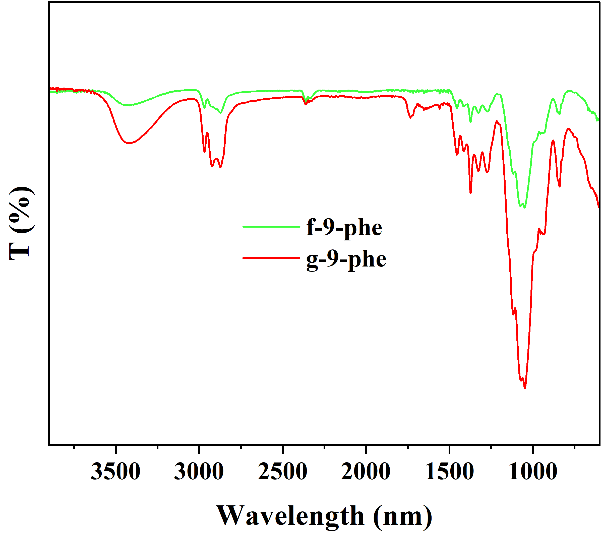
**

**Figure S36** FT-IR spectra of f-9-phe and g-9-phe.


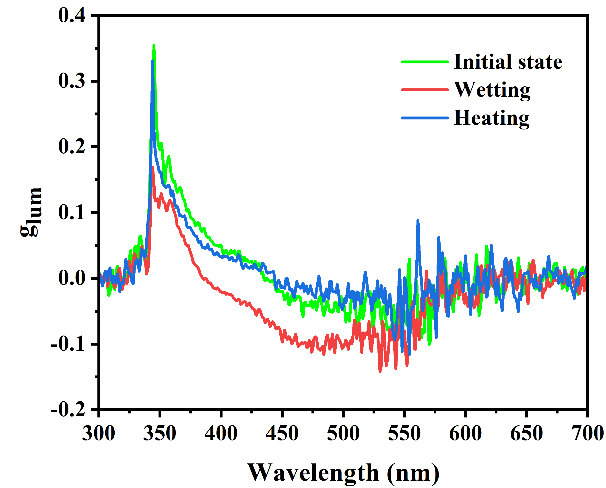


**Figure S37** g_lum_ curve of CPL of g-9-phe.


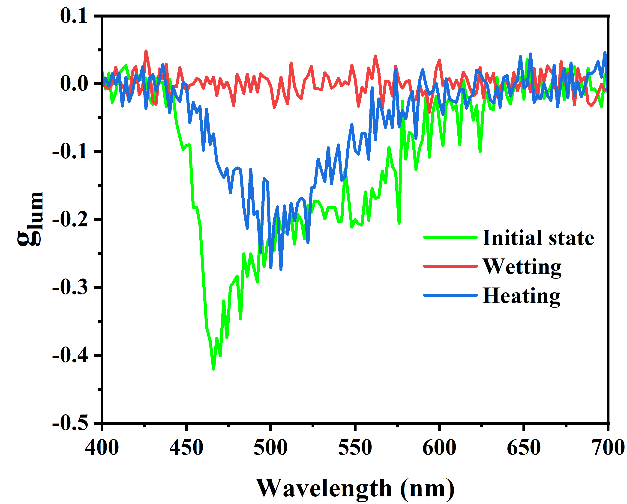


**Figure S38** g_lum_ curve of CPRTP of g-9-phe.


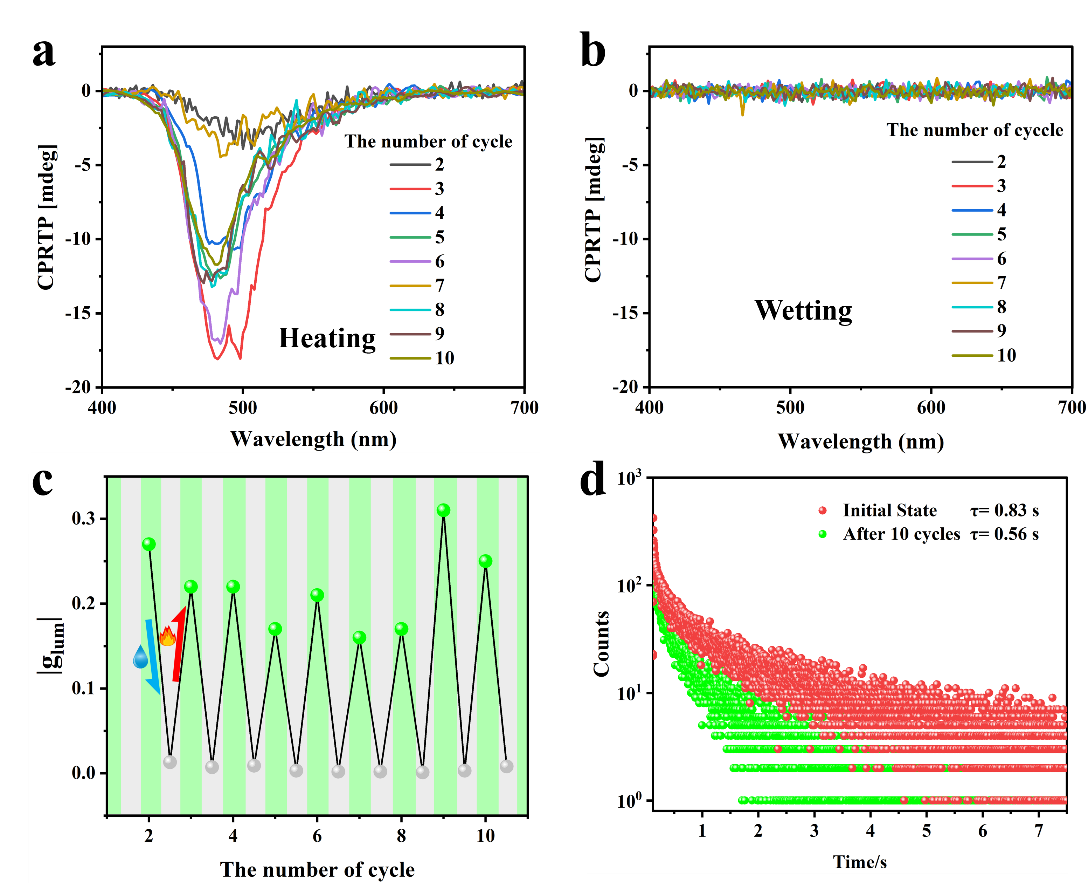


**Figure S39** **a-b)** CPRTP performance of g-9-phe during 10 water/heat stimulus response cycles; **c)** Schematic illustration of the change in asymmetry factor of g-9-phe during 10 water/heat stimulus response cycles; **d)** Lifetime of g-9-phe in its initial state and after undergoing 10 water/heat stimulus response cycles.

**
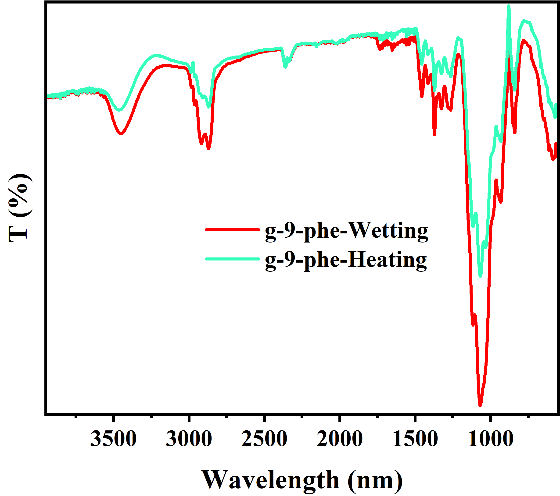
**

**Figure S40** FT-IR spectra of g-9-phe and g-9-phe.

**
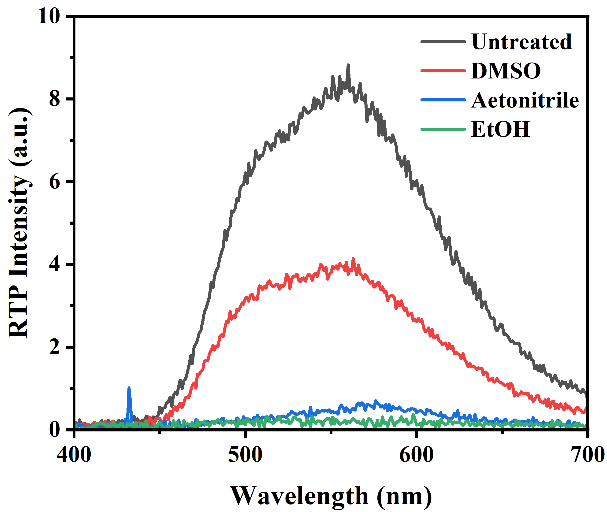
**

**Figure S41** RTP spectra of g-9-phe after 310 nm UV excitation treated with ethanol (EtOH), dimethyl sulfoxide (DMSO), acetonitrile (MeCN), and untreated.


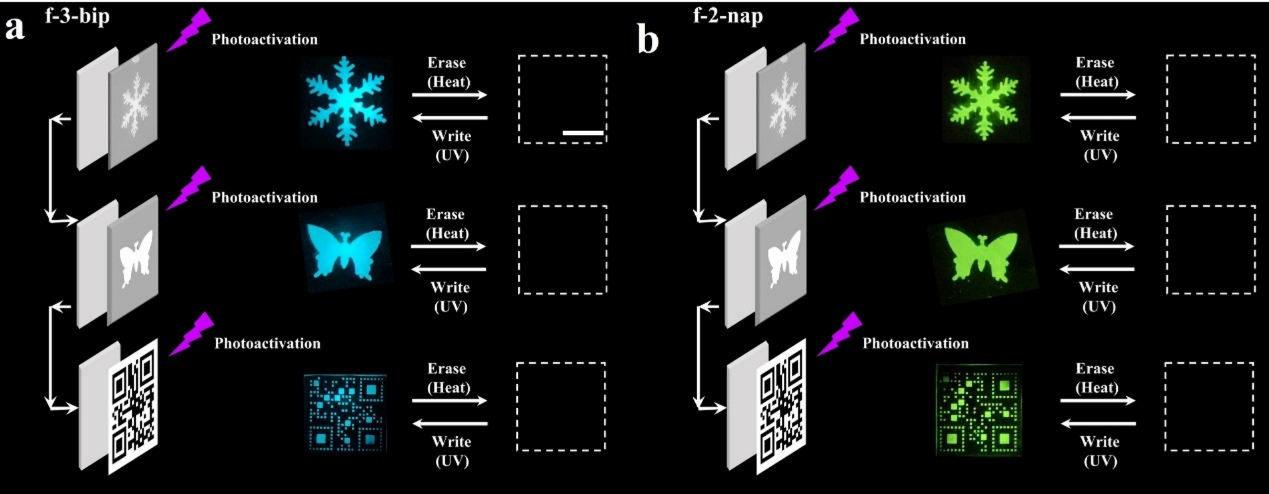


**Figure S42** Demonstration of programmable and reusable afterglow labeling via masked photoprinting technology, scale bar = 1 cm.


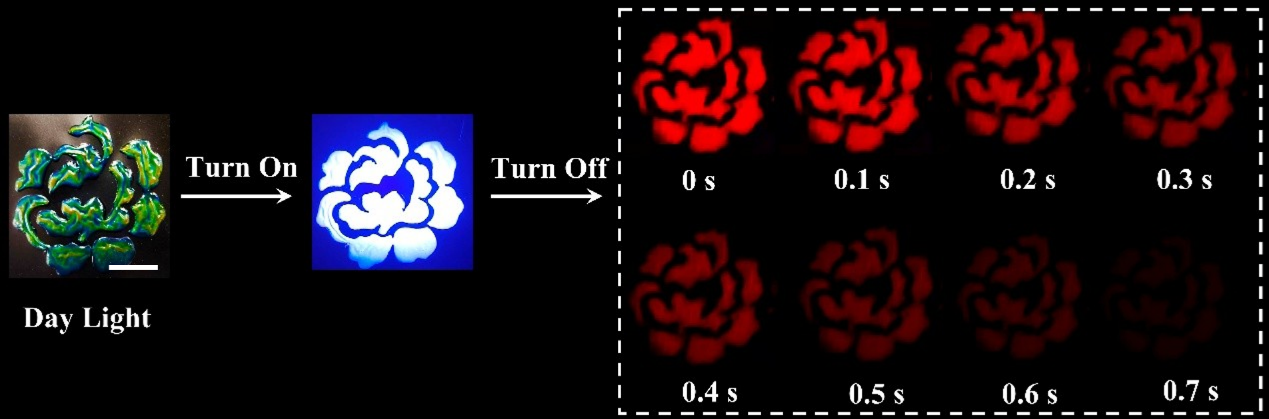


**Figure S43** The delay time of RTP-HPC as the phosphorescent ink, scale bar = 1 cm.

**
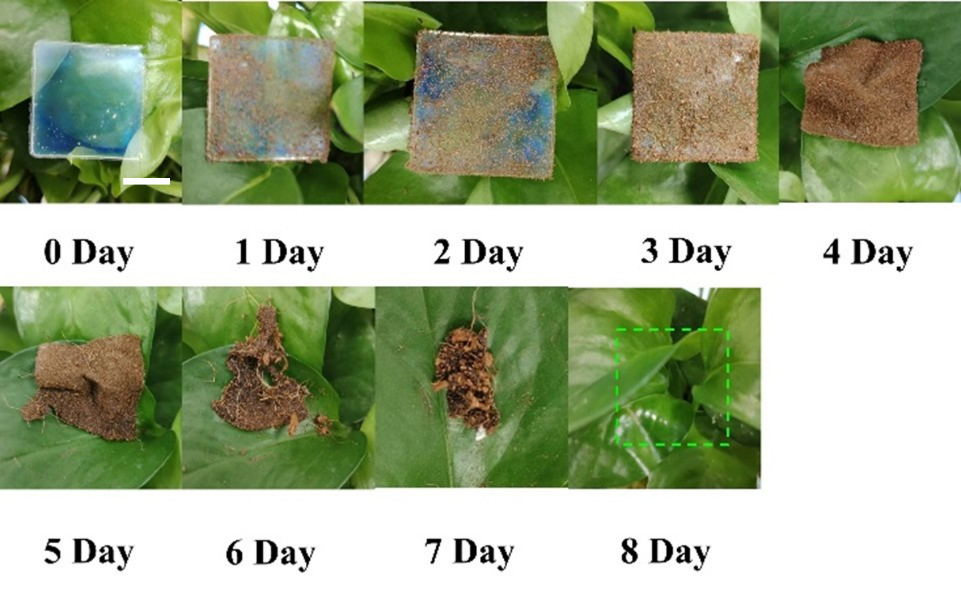
**

**Figure S44** The biodegradation properties of g-9-phe, scale bar = 1 cm.

# **Supporting Table**

**Table S1** Comparison of RTP-HPC with bio-based and photoactivated CPRTP systems.

| Sample | Source | Photoactivated time | Lifetime | **\|**g_lum_**\|** | Reference |
| --- | --- | --- | --- | --- | --- |
| RTP-HPC | HPC | 25 s | 1.57 s | 0.43 | This work |
| P2-CD | CNC/PVA | - | 103 ms | 0.47 | [13] |
| CNC-G-P2 | CNC/Glucose | - | 371 ms | 0.59 | [14] |
| Lig ⊂ CNC | CNC/Lignin | - | 103 ms | 0.21 | [15] |
| NtCOOLi/CMCNa | CMC | - | 936 ms | 0.01 | [16] |
| F_8, 20_ | CNC/PVA | - | 1.73 s | 0.25 | [17] |
| TBB-6OMe/CEC | CEC | - | 184 ms | 0.01 | [18] |
| CNS-PBG | CNC/SiO_2_ | - | 1.09 s | 0.13 | [19] |
| CNC-BI | CNC/Glucose | - | 2.04 s | 0.65 | [20] |
| Cell | Cellulose acetate | - | 561 ms | 0.16 | [21] |
| Phe9-B@CNSF | chitosan | - | 805 ms | 0.29 | [22] |
| PSeA | Polystyrene | 🗸 | 85.9 ms | 0.009 | [23] |
| M3 | PMA | 🗸 | 4.87 ms | 0.07 | [24] |
| S-COOCz | Carbazole acts | 40 s | 600 ms | 0.002 | [25] |
| R/S-CLC-TPB | CLC | 1min | 125 ms | 1.6 | [26] |

# **Reference**

[1] F. Neese, “Software update: The ORCA program system—Version 5.0” *WIREs Comput. Mol. Sci.* **2022**, *12*, e1606.

[2] D. Ao, B. Wang, Y. Wang, Y. Chen, C. Liang, S. An, “Arbuscular mycorrhizal fungi communities and glomalin mediate particulate and mineral-associated organic carbon formation in grassland patches” *Commun. Earth Environ.* **2025**, *6*, 553.

[3] S. Grimme, S. Ehrlich, L. Goerigk, “Effect of the damping function in dispersion corrected density functional theory” *J. Comput. Chem.* **2011**, *32*, 1456–1465.

[4] S. Grimme, “Density functional theory with London dispersion corrections” *WIREs Comput. Mol. Sci.* **2011**, *1*, 211–228.

[5] M. J. Frisch, J. A. Pople, J. S. Binkley, “Self-consistent molecular orbital methods 25. Supplementary functions for Gaussian basis sets” *J. Chem. Phys.* **1984**, *80*, 3265–3269.

[6] F. Weigend, “Accurate Coulomb-fitting basis sets for H to Rn” *Phys. Chem. Chem. Phys.* **2006**, *8*, 1057.

[7] Y. Zhao, D. G. Truhlar, “The M06 suite of density functionals for main group thermochemistry, thermochemical kinetics, noncovalent interactions, excited states, and transition elements: two new functionals and systematic testing of four M06-class functionals and 12 other functionals” *Theor. Chem. Acc.* **2008**, *120*, 215–241.

[8] J. Zheng, X. Xu, D. G. Truhlar, “Minimally augmented Karlsruhe basis sets” *Theor. Chem. Acc.* **2011**, *128*, 295–305.

[9] T. Lu, Q. Chen, “Interaction Region Indicator: A Simple Real Space Function Clearly Revealing Both Chemical Bonds and Weak Interactions**” *Chemistry–Methods* **2021**, *1*, 231–239.

[10] T. Lu, F. Chen, “Quantitative analysis of molecular surface based on improved Marching Tetrahedra algorithm” *J. Mol. Graph. Model.* **2012**, *38*, 314–323.

[11] W. Humphrey, A. Dalke, K. Schulten, “VMD: Visual molecular dynamics” *J. Mol. Graph.* **1996**, *14*, 33–38.

[12] T. Lu, F. Chen, “Multiwfn: A multifunctional wavefunction analyzer” *J. Comput. Chem.* **2012**, *33*, 580–592.

[13] M. Xu, X. Wu, Y. Yang, C. Ma, W. Li, H. Yu, Z. Chen, J. Li, K. Zhang, S. Liu, “Designing Hybrid Chiral Photonic Films with Circularly Polarized Room-Temperature Phosphorescence” *ACS Nano* **2020**, *14*, 11130–11139.

[14] J. Liu, X. Zhou, J. Wei, J. Wu, J. Hu, X. Fang, R. Lan, Y. Tao, Y. Ma, B. Li, H. Yang, Y. Lu, Q. Zhao, “Multi‐Color Circularly Polarized Room‐Temperature Phosphorescence from Processable Chiral Photonic Films” *Adv. Funct. Mater.* **2025**, *35*, 2506911.

[15] M. Cao, Y. Ren, Y. Wu, J. Shen, S. Li, Z.-Q. Yu, S. Liu, J. Li, O. J. Rojas, Z. Chen, “Biobased and biodegradable films exhibiting circularly polarized room temperature phosphorescence” *Nat. Commun.* **2024**, *15*, 2375.

[16] J. You, C. Yin, S. Wang, X. Wang, K. Jin, Y. Wang, J. Wang, L. Liu, J. Zhang, J. Zhang, “Responsive circularly polarized ultralong room temperature phosphorescence materials with easy-to-scale and chiral-sensing performance” *Nat. Commun.* **2024**, *15*, 7149.

[17] S. Jia, B. Yang, J. Du, W. Tang, J. Gong, “Circularly Polarized Ultralong Room‐Temperature Phosphorescence and Multi‐Color Afterglow via the Stepwise Energy Transfer” *Adv. Opt. Mater.* **2025**, *13*, e01631.

[18] J. You, R. Tian, C. Yin, J. Wang, J. Zhang, J. Zhang, “Organic Circularly Polarized Room-Temperature Phosphorescence Toolbox with Excellent Practicality and Functionality” *ACS Nano* **2025**, *19*, 38219–38230.

[19] D. Zhang, H. Zheng, X. Ma, L. Su, X. Gao, Z. Tang, Y. Xu, “On‐Demand Circularly Polarized Room‐Temperature Phosphorescence in Chiral Nematic Nanoporous Silica Films” *Adv. Opt. Mater.* **2022**, *10*, 2102015.

[20] X. Nie, Y. Zhang, B. Wu, Z. Ye, F. Gao, Y. Chen, C. Wang, D. Zhu, P. Alam, Z. Qiu, B. Z. Tang, “Dynamic Chirality in Nature-Inspired Photonic Crystal Films: Ultralong Room Temperature Phosphorescence and Stimuli-Responsive Circularly Polarized Luminescence” *ACS Nano* **2025**, *19*, 11221–11229.

[21] K. Jin, C. Yin, J. You, H. Diao, J. Wang, K. Zhu, J. Zhang, J. Zhang, “Large-scale and flexible circularly polarized room temperature phosphorescence with a high dissymmetry factor and chiral sensing” *Innov. Mater.* **2024**, *2*, 100096.

[22] H. Wang, W. Zhang, J. Huang, Y. Zhang, H. Li, L. Huang, W. Li, F. Zhang, G. Qing, “Ultra‐Stable, Long‐Lived, and Multicolor Circularly Polarized Room‐Temperature Phosphorescence Enabled by Shrimp‐Derived Chitosan Nanocomposite Chemistry” *Adv. Funct. Mater.* **2025**, e27613.

[23] S. Fu, Y. Chen, Y. Xie, Z. Li, “Photoactivated Circularly Polarized Room‐Temperature Phosphorescence from Phenoselenazine Derivative and Its Application in Information Security^†^” *Chin. J. Chem.* **2024**, *42*, 2499–2506.

[24] C. Liu, H. Li, Y. Chen, D. Xu, Y. Cheng, “Circularly polarized room-temperature phosphorescence based on chiral co-assembled helical nanofiber from chiral co-assembled liquid crystal co-polymer” *Chem. Eng. J.* **2024**, *486*, 150442.

[25] H. Li, H. Li, W. Wang, Y. Tao, S. Wang, Q. Yang, Y. Jiang, C. Zheng, W. Huang, R. Chen, “Stimuli‐Responsive Circularly Polarized Organic Ultralong Room Temperature Phosphorescence” *Angew. Chem. Int. Ed.* **2020**, *59*, 4756–4762.

[26] Y. Zhang, Y. Wang, J. Wu, A. Shao, J. Liu, S. Liu, B. Li, Y. Ma, Q. Zhao, “Enhanced Photoactivated Circularly Polarized Afterglow with High Dissymmetry Factor and Tunable Emission” *Adv. Funct. Mater.* **2025**, *35*, 2424404.
